# Meta-Assessment of Metformin Absorption and Disposition Pharmacokinetics in Nine Species

**DOI:** 10.3390/ph14060545

**Published:** 2021-06-07

**Authors:** Yoo-Seong Jeong, William J. Jusko

**Affiliations:** Department of Pharmaceutical Sciences, School of Pharmacy and Pharmaceutical Sciences, State University of New York at Buffalo, Buffalo, NY 14214, USA; yooseong@buffalo.edu

**Keywords:** metformin, pharmacokinetics, absorption, disposition, allometric scaling, minimal physiologically based pharmacokinetic modeling

## Abstract

The objective of this study was to systematically assess literature datasets and quantitatively analyze metformin PK in plasma and some tissues of nine species. The pharmacokinetic (PK) parameters and profiles of metformin in nine species were collected from the literature. Based on a simple allometric scaling, the systemic clearances (CL) of metformin in these species highly correlate with body weight (BW) (R^2^ = 0.85) and are comparable to renal plasma flow in most species except for rabbit and cat. Reported volumes of distribution ($V_{SS}$) varied appreciably (0.32 to 10.1 L/kg) among species. Using the physiological and anatomical variables for each species, a minimal physiologically based pharmacokinetic (mPBPK) model consisting of blood and two tissue compartments (Tissues 1 and 2) was used for modeling metformin PK in the nine species. Permeability-limited distribution (low $f_{d1}$ and $f_{d2}$) and a single tissue-to-plasma partition coefficient ($K_{p}$) value for Tissues 1 and 2 were applied in the joint mPBPK fitting. Nonlinear regression analysis for common tissue distribution parameters along with species-specific CL values reasonably captured the plasma PK profiles of metformin across most species, except for rat and horse with later time deviations. In separate fittings of the mPBPK model to each species, Tissue 2 was considered as slowly-equilibrating compartment consisting of muscle and skin based on in silico calculations of the mean transit times through tissues. The well-fitted mPBPK model parameters for absorption and disposition PK of metformin for each species were compared with in vitro/in vivo results found in the literature with regard to the physiological details and physicochemical properties of metformin. Bioavailability and absorption rates decreased with the increased BW among the species. Tissues such as muscle dominate metformin distribution with low permeability and partitioning while actual tissue concentrations found in rats and mice show likely transporter-mediated uptake in liver, kidney, and gastrointestinal tissues. Metformin has diverse pharmacologic actions, and this assessment revealed allometric relationships in its absorption and renal clearance but considerable variability in actual and modeled tissue distribution probably caused by transporter differences.

## 1. Introduction

Metformin is a biguanide compound that was derived from *Galega officinalis* (French lilac) having an abundant content of the hypoglycemic substance guanidine [1,2]. Although the specific compound metformin was first synthesized in 1920s [3,4] and its use was later replaced by the breakthrough discovery of insulin in the same era [5], experience with guanidine and diguanides for treating diabetes led to development of phenformin [6], buformin [7], and metformin (Glucophage was named by Jean Sterne) in the 1950s [8,9]. However, after phenformin and buformin were withdrawn from the market in most countries due to their high risks of lactic acidosis [10], metformin has been recommended as the first-line oral medication for treatment of type 2 diabetes by the American Diabetes Association (ADA) and the European Association for the Study of Diabetes (EASD) since 2009 [11]. In addition to its antihyperglycemic effect, the United Kingdom Prospective Diabetes Study (UKPDS) reported long-term metabolic effects and reduced cardiovascular risk with metformin [12]. Metformin is increasingly recognized as a potential anticancer agent due to a reduced cancer incidence in diabetic patients treated with the drug [13,14,15], and recently, patients taking metformin were associated with a reduced risk of COVID-19-related mortality [16,17]. The history of metformin from its discovery in the 18th century to clinical uses for over 60 years is well-summarized in the literature [18], and the drug is now widely used with more than 500 million prescriptions in United States with growth trends since 2000 [19].

Although its mechanism of action remains unclear even with the long-term experiences of clinical use, the blood glucose-lowering effect of metformin is attributed to several tissues. The major mode of its pharmacological action is decreased hepatic gluconeogenesis by modulation of mitochondrial enzymes and by the antagonistic regulation of the glucagon signaling pathway [20,21]. In vitro studies in rodents showed that metformin improved insulin sensitivity of muscle by increasing the expression and activity of insulin receptors, leading to increased insulin-dependent glucose uptake in cells [22,23,24]. In addition, increased glucose uptake into the intestinal mucosa [25] and reduced intestinal glucose absorption [26] were observed in metformin-treated rats. A compartmental model accounting for amounts of metformin in plasma, liver, muscle, and gastrointestinal (GI) tract provided an insightful quantitative assessment of glucose-lowering effects that differ with route of administration in diabetic rats [27]. An alternative model for glucose production inhibited by metformin distributed to a biophase compartment described its blood-glucose lowering effect in diabetic rats at different doses [28]. Pharmacodynamic studies of metformin in rat or man describing its effects over a range of doses are few.

Oral absorption of metformin from the immediate-release dosage forms is incomplete in man, with an estimated population mean of 55% for bioavailability [29]. Metformin, a strong base with pKa = 11.8 [30], is present mostly as a cationic form at biologically relevant pH ranges and has a high water solubility larger than 100 mg/mL [31]. Its hydrophilicity (log P = −1.43) [32] is associated with the low intestinal [33] and cell membrane permeability [34], which is recognized as a primary limiting step for metformin oral absorption and accounts for its insignificant metabolism in the liver. Metformin is mainly eliminated by renal excretion mediated by transporters, including organic cation transporters (OCT1 and OCT2) and multidrug and toxin extrusion proteins (MATE1 and MATE2) [35]. Dosage adjustments are thus necessary for patients with severe renal dysfunction [36,37,38]. In healthy subjects, a longer half-life of metformin in erythrocytes (23.4 h) was observed compared to plasma (2.7 h) [39], but this may be attributed to the time frames assessed. Similarly, a longer half-life of metformin was also observed in following urinary excretion rates (9.3 to 19.0 h) [37,40]. Metformin concentrations in tissues have been measured using radiolabeled compounds (i.e.,^14^C-label for mouse and ^11^C-label for man), as well as use of PET (Positron Emission Tomography) scanning [41,42]. However, a limited number of time points/tissues, potential involvement of its metabolite(s), and indistinguishability of intra- and extracellular and the intravascular fractions using PET are limiting factors. The drug exhibits either poly-exponential or power function disposition with uncertainties regarding its distribution kinetics, the terminal half-life, and recoveries in urine.

When only pharmacokinetic (PK) profiles in plasma are available, minimal physiologically based pharmacokinetic (mPBPK) models allow a rational interpretation of plasma and expected tissue PK by the incorporation of physiological and anatomical elements into PK analyses [43]. In line with this, allometric scaling is often utilized to relate drug disposition processes to body size [44]. The renal clearance of metformin was reported to have a simple allometric relationship with body weight (BW) with a common exponent of 0.75 [45]. However, traditional allometric scaling assessing PK parameters in relation to BW (i.e., $Y=a\cdot BW^{b}$) among species is considered empirical [46]. Thus, integrating allometric features into mPBPK modeling is useful for interspecies scaling of PK characteristics. Previously, mPBPK models were successfully applied for across-species fitting and scaling of full PK profiles of moxifloxacin [43], dexamethasone [47], and several monoclonal antibodies [48]. Despite abundant PK data for metformin in various species from its wide use in clinical/veterinary and experimental settings for over 60 years, there has been only limited quantitative analysis assessing interspecies relationships regarding PK properties of metformin [49,50].

In reviewing the extensive literature on metformin PK, we first assessed its clearance (CL) in relation to BW and renal plasma flow (RPF) across species using traditional allometry. We then employed mPBPK models to quantitatively analyze metformin PK in all nine species. Joint fittings using mPBPK models that adopted the physiological and anatomical properties of each species were applied to obtain common PK parameters with conserved features across the species seeking a minimal number of parameters that could describe all data. As a third step, a mPBPK model structure based on in silico-predicted tissue constituents was used to further determine and compare the species-dependent PK parameters related to absorption and disposition of metformin for each of the nine species. This systematic review of literature datasets and quantitative analyses of metformin PK in plasma and some tissues of all available species should be useful for further evaluation of the pleiotropic pharmacological effects of metformin.

## 2. Results

### 2.1. Allometric Scaling

The PK parameters of metformin after the intravenous (IV) and oral doses found in nine species are summarized in Table 1. The raw data for metformin PK in dog were provided by Johnston et al. [51], and all others were digitized for the current analysis. When PK parameters in any species were found to be variable among multiple studies (e.g., CL values ranging from 12.7 to 81.7 mL/min/kg in mouse and from 6.0 to 42.8 mL/min/kg in rat; Supplementary Table S1), PK data were selected on the basis of the richness of sampling time points, duration of sampling periods, details of study design, and alignment of CL values with basic allometric scaling. It is noted that, due to variable PK profiles depending on the oral doses in rats in previous literature that included the same IV doses (50, 100, and 200 mg/kg) [52], another oral PK profile at a similar experimental setting [53] was considered for the individualized analysis.

**Figure 1:**
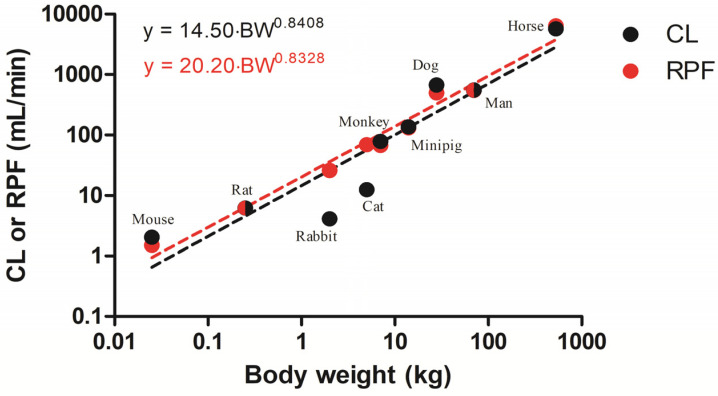
Allometric relationships between literature-reported metformin CL (black) and renal plasma flow (RPF) values (red) and body weights of 9 species (Table 3, see below). Except for rabbit and cat, the systemic clearance of metformin was comparable to RPF. Lines were fitted to the indicated allometric relationships ($y=a\cdot BW^{b}$, where BW is the body weight).

As shown in Figure 1, the reported CL values for metformin showed a simple allometric relationship with BW of the nine species (R^2^ = 0.85). Despite this reasonable correlation, the systemic CL values of metformin in rabbit and cat deviate from the fitted relationship (with a factor of 6.33 and 4.49). Except for these two species, CL values were comparable with RPF, indicating a high extraction ratio of metformin in the kidney, and were consistent with evidence that metformin is mainly eliminated via renal excretion [40,73].

### 2.2. Joint Fittings of IV Profiles

**Figure 2:**
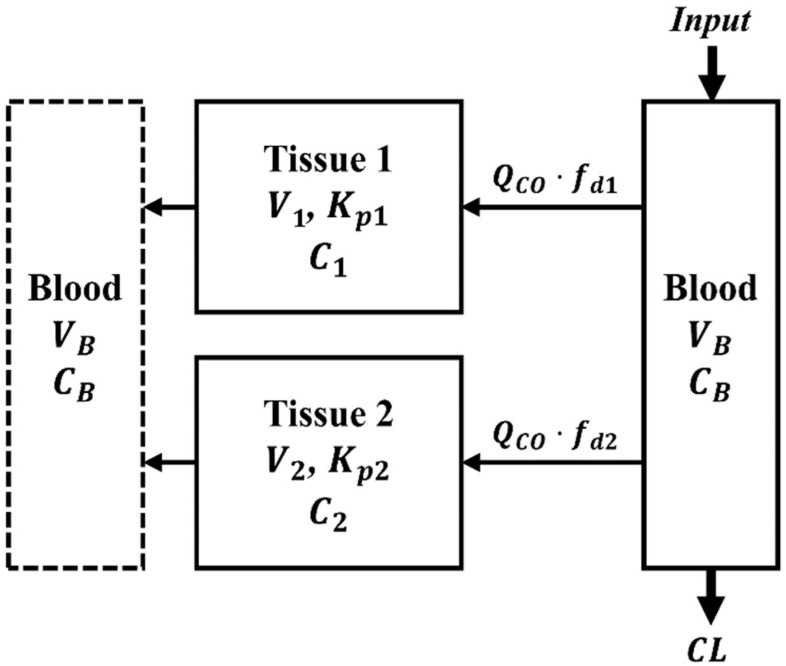
The minimal physiologically based pharmacokinetic model with two tissue compartments. A single $K_{p}$ value ($K_{p1}=K_{p2}$) was used in the joint fitting, while the cardiac output ($Q_{CO}$) was divided into tissue blood flow to each compartment ($Q_{1}+Q_{2}=Q_{CO}$) in the separate fittings. Symbols are defined in the text and in Table 2 and Table 5 (see below).

**Figure 3:**
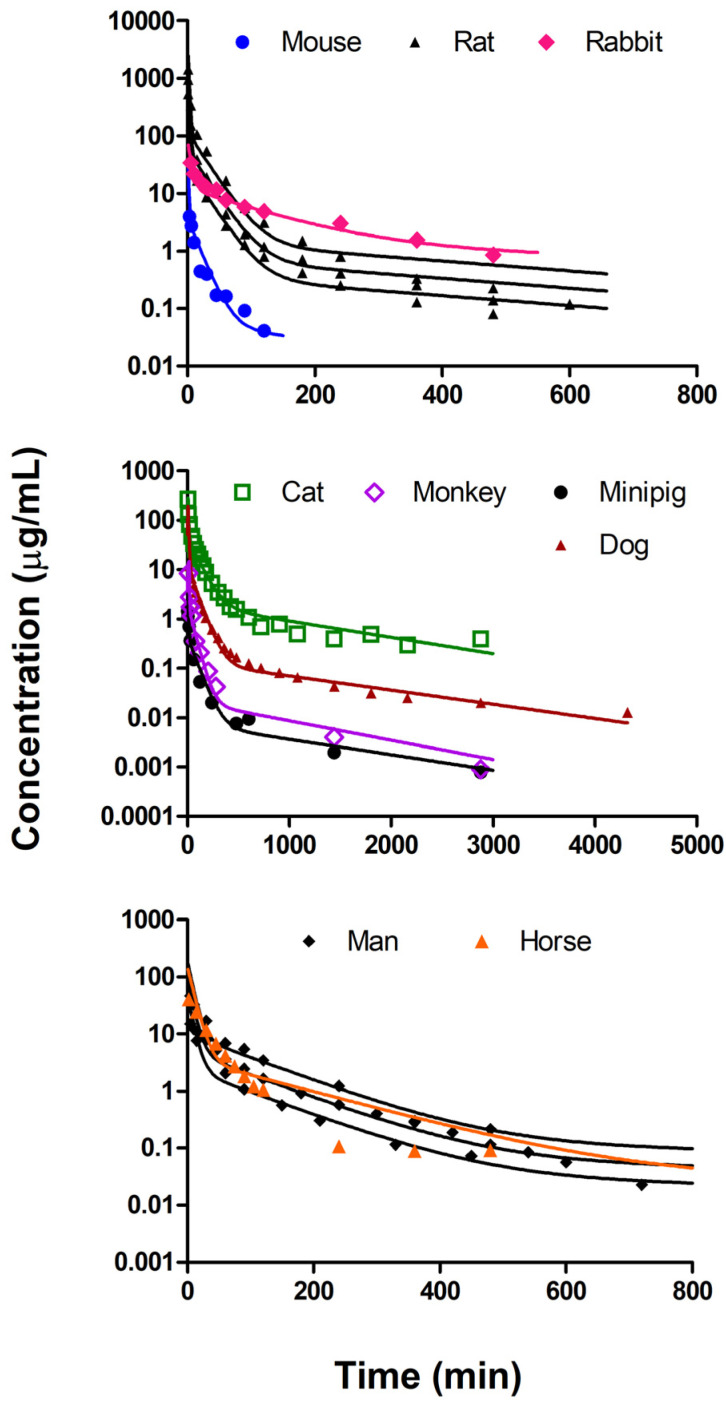
Simultaneous fitting of metformin pharmacokinetics dosed intravenously in 9 species to the minimal physiologically based pharmacokinetic model in Figure 2. Fitted profiles are in separate panels for visual clarification for the indicated species. The doses and fitted parameters are listed in Table 1 and Table 2. The three curves for man are for different doses from three studies.

The metformin PK data were first jointly analyzed using the common parameters across the species adopted in the mPBPK model ($K_{p}$, $f_{d,total}$, $f_{d1}$, $f_{d2}$, and $f_{t}$), along with the individual optimized CL for each species. Figure 2 shows the mPBPK model structure used for metformin PK and the differential equations for the current analysis are described in Methods section. As shown in Figure 3, the plasma concentrations across species ranged from about 1000 to 0.001 µg/mL, with rapid and slower phases often extending to 72 h. A wide range of IV doses were given (Table 1), and the plasma concentrations were not normalized for the dose. The mPBPK model anticipates triexponential disposition profiles that appear in all species. Consistent with its hydrophilicity (log P = −1.43) [32] and low passive permeability [74], the assumption of perfusion-limited distribution (i.e., $f_{d1}+f_{d2}=1$) did not adequately capture the PK data in our preliminary mPBPK modeling. Thus, it was reasoned that the sum of the two fractional distribution parameters would be less than 1 and conserved across the species in the joint fitting approach (Equation (7)). In addition, a consideration of different $K_{p}$ values for each tissue compartment ($K_{p1}$ and $K_{p2}$) did not work well, leading to unreliable estimates (extremely high CV%) for parameters $K_{p1}$, $K_{p2}$, and $f_{t}$. As a result, the PK profiles were reasonably captured by the current mPBPK model, except for rat and horse, with later time deviations. However, the late PK data in horses were difficult to digitize and the mouse data lacked a terminal phase. The three IV dose profiles for man came from three different studies (Table 1) and all were very well-captured. Despite these divergent fittings, CV% values for the distribution parameters of the joint mPBPK model were all less than 10% (Table 2). The fitted CL for each species from the joint fitting are listed in Table 3 (all CV% less than 15%). Based on the single $K_{p}$ assumption, the physical volume of Tissue 1 (0.16∙BW) was smaller than Tissue 2 (0.77∙BW). The $f_{d,total}$ is 0.0457, which implies that 4.57% of the drug is subjected to extraction from the blood during each passage through tissues, as the total distributional clearance $CL_{D}$ is $Q_{CO}\cdot f_{d,total}$ (Equation (2)). The distributional clearance to Tissue 1 or 2 can be calculated by multiplying either $f_{d1}$ or $f_{d2}$ by the cardiac output ($Q_{CO}$) of each species, indicating that Tissue 1, which has a shorter MTT, is more rapidly equilibrated than Tissue 2 ($MTT_{1}=V_{1}K_{p}/\left(Q_{CO}f_{d1}R_{b}\right)$ and $MTT_{2}=V_{2}K_{p}/\left(Q_{CO}f_{d2}R_{b}\right)$). The overall $K_{p}$ value is 1.29, which is midrange to many $V_{SS}$ values found by noncompartmental analysis (NCA) (0.321 to 2.26 L/kg, except for dogs), as listed in Table 1. The $V_{SS}$ value for dogs calculated by NCA using the averaged concentration–time data was 10.1 L/kg, which was accompanied by the underestimation of the systemic CL for dogs in the joint fitting (9.01 mL/min/kg, compared to the reported CL of 24.1 mL/min/kg) within a restricted range of distribution parameters. Due to the model prediction showing the terminal phase with a longer half-life than the limited observations in mouse (Figure 3), divergent NCA and jointly fitted CL values were observed (Table 3).

### 2.3. Individual Oral and IV Fittings

**Figure 4:**
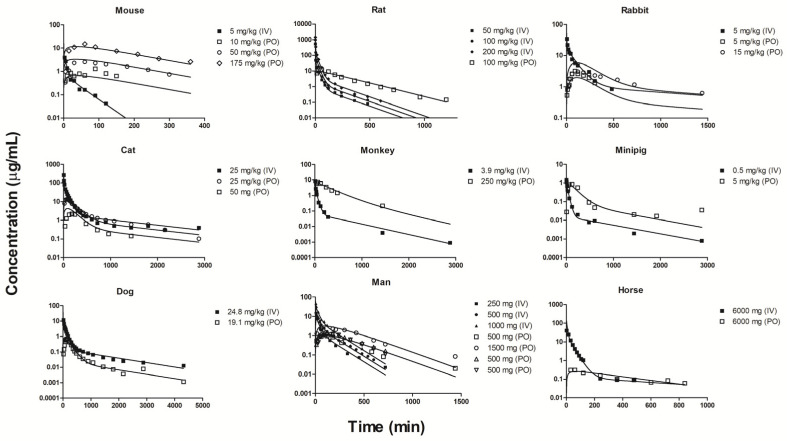
Fitting of intravenous and oral pharmacokinetics of metformin individually for 9 species to the mPBPK model. The slowly-equilibrating compartment (Tissue 2) was assumed to consist of skin and muscle (i.e., $Q_{2}=Q_{SK}+Q_{MU}$, $V_{2}=V_{SK}+V_{MU}$). Fitted parameters are listed in Table 5.

In order to investigate potential differences in PK variables for metformin across the species, we carried out further model fittings of both IV and oral PK profiles separately for each of the nine species. Having multiple PK profiles in each species adds greater certainty in resolving model parameters. As shown in Figure 4, all PK profiles were well-captured. Tissue lumping theories [75,76,77,78] state that the major organ(s) composing the rapidly and slowly equilibrating compartments (Tissues 1 and 2) that, in turn, govern the exponential components/shapes of the systemic PK may not be identifiable without information on tissue PK. However, muscle was included in Tissue 2, since the $f_{t}$ estimate of 0.172 from the joint fitting (Table 2) was small. Using $S_{eff}$ values in a WB-PBPK structure for rats [79], the tissue permeability coefficient P was estimated to be 0.0102 × 10^−6^ cm/s with a CV% of 29.4% (R^2^ = 0.98). As a result, in silico calculations (Table 4) consistently indicate that skin may be the kinetically largest tissue with the longest MTT for a variable range of permeability coefficients. A rule of thumb for lumping kinetically large tissues suggests that tissues within a two-fold difference in MTT may be lumped into a single compartment [75], which was satisfied by MTT values calculated from the in vitro PAMPA P for muscle and skin (Table 4). The brain, the second-largest tissue based on the case of fitted P that satisfies the acceptance condition with a factor of 1.62, was not included in Tissue 2, considering the variable $K_{p}$ values for rat brains (cf. in vivo $K_{p}$ ranged from 0.2 to 1.48 (Table 6, see below)), compared to an in silico value of 0.86 (Table 4), where the involvement of the efflux transport of metformin by P-glycoprotein [80] may reduce $K_{p}$ and, thus, MTT. Based on these considerations, the mPBPK model in our separate fitting approach utilizes the blood flow rate and anatomical volume of skin and muscle to define Tissue 2, with the fractional tissue volume ($f_{t}=V_{2}/\left(BW-V_{B}\right)$) ranging from 0.472 to 0.678 and blood flow ($Q_{2}/Q_{CO}$) from 0.217 to 0.563 (Supplementary Table S2).

The IV and oral PK profiles were reasonably captured (Figure 4) in all species with the fitted parameters listed in Table 5. It is noted that the 48-h time point in the oral PK profile in minipig was excluded in the current analysis as an obvious outlier as inclusion produced parameter estimates with an unreliable CV% and an extremely shallow terminal phase. It should be noted that the terminal phase slope in flip-flop kinetics is dependent on the total escaping rate from the absorption site ($k_{a}$), not $F\cdot k_{a}$. The half-lives of $k_{a}$ in mouse, rat, and man are 1.85, 2.82, and 1.74 h (Table 5), which are longer than model-predicted terminal phase half-lives, leading to flip-flop kinetics in these species (Figure 4). As shown in Table 5, the $f_{d1}$ values were smaller than $f_{d2}$ for each species, while $K_{p1}$ was smaller than $K_{p2}$ except for rat, indicating that $MTT_{2}$ is longer than $MTT_{1}$. Except for $f_{d1}$ for mouse and $K_{p2}$ for horse, the largest CV% of the primary estimates was 93.5%, but the others were quite small (Table 5), indicating reasonable model performances.

**Figure 5:**
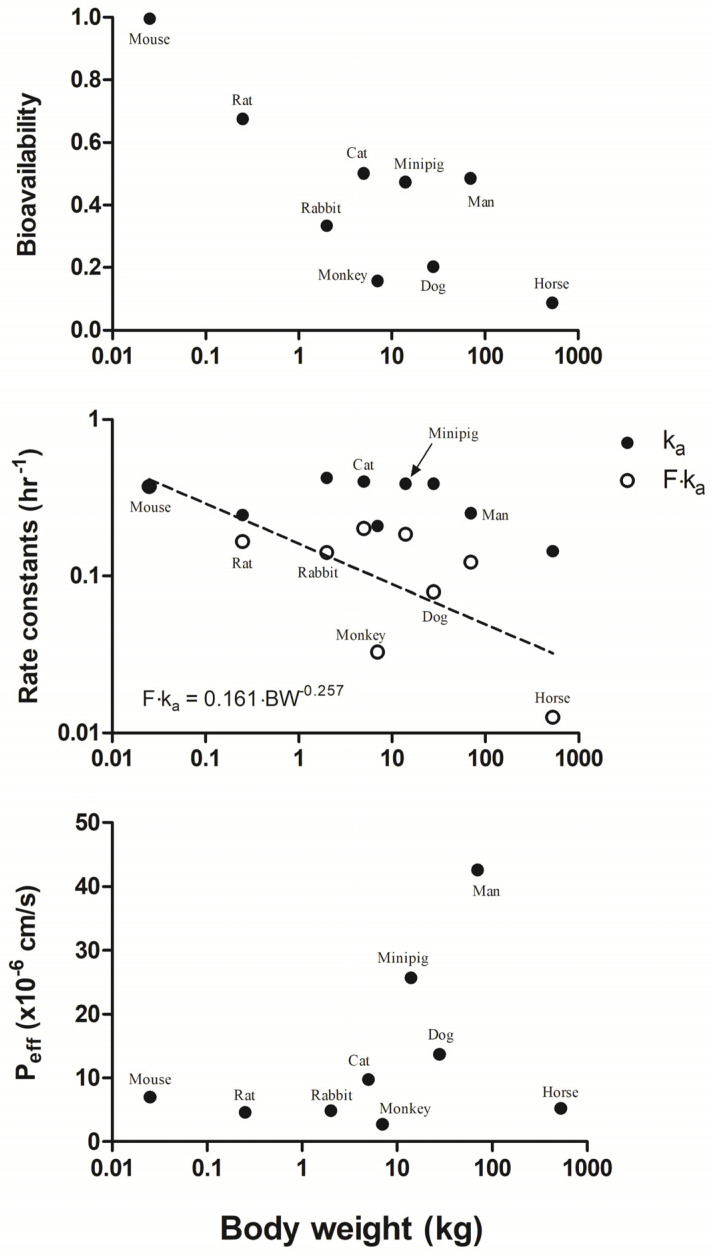
Fitted pharmacokinetic parameters related to oral absorption of metformin in relation to body weights, including bioavailability (F), absorption rate constants ($k_{a}$ and $F\cdot k_{a}$), and intestinal effective permeability ($P_{eff}$). The fitted parameters for each species are listed in Table 5.

The estimated PK parameters related to oral absorption of metformin from the separate fittings are shown in Figure 5. The model-fitted bioavailability F for the nine species showed a decreasing trend with increasing BW. While the $k_{a}$ values were statistically independent of BW (i.e., allometric exponent with statistically insignificant difference from 0), the effective absorption rate constant ($F\cdot k_{a}$) was highly correlated with BW with an allometric exponent of 0.257 (R^2^ = 0.54). This is consistent with a common allometric exponent of 0.25 found for the rate constants [44]. According to the Biopharmaceutics Classification System (BCS) [83], metformin is considered a Class III drug based on its high water solubility as a hydrochloride salt (i.e., >100 mg/mL) [31] and low intestinal effective permeability (e.g., $P_{eff,rat}$ = 0.27 × 10^−4^ cm/s) [33]. Based on the assumption of instantaneous solubilization of metformin into the fluid volumes of the stomach and small intestine ($V_{Lumen}$ values summarized in Supplementary Table S2), $Dose/V_{Lumen}$ was estimated to be far below 100 mg/mL for the maximum dose of 175 mg/kg in mouse (i.e., 10.9 mg/mL). Therefore, expecting that intestinal permeability is the major determinant governing metformin absorption, the effective permeability ($P_{eff}$) was estimated by considering the GI tract as a cylinder (i.e., $F\cdot k_{a}=\frac{2P_{eff}}{R},$ where R is the gut radius). These were particularly higher in minipig, man, and dog than other species.

### 2.4. Tissue Distribution of Metformin

Part of this literature review included the assessment of actual tissue-to-plasma partition coefficients of metformin. All available measurements are listed in Supplementary Table S3, with means and ranges summarized in Table 6. In vivo data could be mostly found for rats and mice, except for protein binding and erythrocyte distribution. The drug appeared to sequester most highly in the GI tract, kidney, and liver, with tissue-to-plasma ratios of 3 or greater. Large values are expected in kidney owing to the robust transport mechanism and probable inclusion of tubular fluid with excreted drug in the measurements. The high ratios in the liver and kidney were not anticipated when using the tissue $K_{p}$ prediction methods (Table 4) for metformin in rats, as these methods do not account for the role of transporters. Muscle is the dominant tissue in the body by weight and has low tissue-to-plasma ratios of 0.6 to 1.0 that are in accordance with the predicted value (0.79). This likely explains the overall low $K_{p}$ and $V_{SS}$ values found when fitting mouse and rat data using the mPBPK model (Table 5). Using the in vivo tissue $K_{p}$ values listed in Table 6, along with the muscle $K_{p}$ assumed to be the same for the unavailable tissues, the calculated $V_{SS}$ ($V_{SS}=V_{B}+\sum V_{T,i}K_{p,i}$) [84] was 2140 mL/kg for mouse and 777 mL/kg for rat (Supplementary Table S4), comparable to the observed $V_{SS}$ of 1840 mL/kg for mouse and 586–693 mL/kg for rat (Table 1).

## 3. Discussion

Metformin is widely used as a first-line agent for the treatment of type 2 diabetes. Its antihyperglycemic effect is mainly achieved by decreasing hepatic gluconeogenesis and glycogenolysis and by increasing the insulin sensitivity of muscle tissue [85,86]. Its advantages include insignificant risks of clinical hypoglycemia, which is a serious side effect caused by antidiabetic agents such as insulin and sulfonylureas [87]. In addition, metformin reduces plasma triglycerides and low-density lipoproteins (LDL) [88,89,90,91], which may decrease the risk of cardiovascular disease. The drug has received considerable attention due to a reduced cancer incidence in diabetic patients treated with metformin [13,14,15]. Inhibition of mitochondrial complex I activity has been proposed to be the mechanism of the antitumor effect [92,93] that could be mostly observed at supra-pharmacological concentrations of metformin (i.e., IC50 of 19–66 mM) [94,95], while the blood glucose lowering effect was mediated by increased mitochondrial respiration and fission at clinically relevant concentrations (e.g., 75 µM) [96]. Other beneficial effects of metformin reported are weight reduction [97], delay of aging [98,99], and reduced risk of COVID-19-related mortality [16,17]. In contrast, metformin use may cause adverse effects such as lactic acidosis, hepatotoxicity, acute pancreatitis, and vitamin B12 deficiency [100]. Due to these pleiotropic pharmacological and toxicological effects of metformin, this modeling analysis was pursued to gain improved understanding of its PK and tissue distribution properties among various species. Metformin PK were found in nine species owing to its wide use in the treatment of diabetes in clinical and veterinary settings for over 60 years. The PubMed search (last accessed 1 May 2021) provided 1733 citations since 1969 using the key words ‘metformin’ and ‘pharmacokinetics’ and 717 citations since 2010 with the topic of ‘the pharmacokinetics of metformin’.

Metformin is well-known to be mostly or entirely eliminated via urinary excretion [40,73]. Though there has been some evidence of incomplete urinary recovery of metformin (e.g., approximately 80%) after IV doses in man [36,37] and rats [52,53,101], the systemic CL was comparable with RPF in most species, except for rabbits and cats (Figure 1). Since the glomerular filtration rate (essentially $f_{up}\cdot GFR$) is much smaller than RPF (Supplementary Table S2), the concordance between CL and RPF implies significant active secretion of metformin by the kidney. While hyperthermia (e.g., over 41 °C) is associated with decreased GFR and RPF [102], the RPF of relatively hyperthermic rabbits (39.1 °C) [103] was aligned with the fitted trendline between BW and RPF (Figure 1). This is consistent with the assessment that body temperature may be not a crucial term for allometric scaling across the mammalian species [104,105]. It is evident in the literature that renal transporters including organic cation transporters (OCT1 and OCT2) and multidrug and toxin extrusion proteins (MATE1 and MATE2) are involved in the active secretion of metformin [35]. Based on a quantitative proteomics approach, related transporters (e.g., OCT2 and MATE1) are highly expressed in the kidney, which were generally comparable among several species (e.g., mouse, rat, monkey, and man), arguing for the case that transporters are conserved [106]. While rabbits exhibited higher $CL_{R}$ particularly for anionic drugs [45], which may be related to a higher extent of organic anion transporters (e.g., rbOAT1 and rbOAT3) compared to OCT transporters [107], discordant CL and RPF in rabbits (Figure 1) may be related to a lower affinity of metformin to rbOCT2 (e.g., IC50 value of 808 µM) than hOCT2 (339 µM) [108]. However, a previous PBPK model incorporating an electrogenic component for OCT1 and OCT2 transport suggested the plasma concentration profiles of metformin may be sensitive to transport activity mainly by MATE1/2 in the kidney [109,110]. Therefore, further studies to explain the systemic CL smaller than RPF in rabbit and cat with regard to the differential expression/activities in the apical membrane transport of metformin in their kidneys would be interesting.

As shown in Figure 3, the joint fitting of the mPBPK model reasonably captured the observed PK profiles for metformin in the nine species. One advantage of this approach is that fitting all profiles together allows the data to be shared in resolving the model parameters. The data from mice were rather limited, while most other species had more extensive measurements over longer times. Based on the in vitro PAMPA P of metformin (0.5 × 10^−6^ cm/s) [74], the apparent PAMPA P coefficient (i.e., $f_{up}P/R_{b}$ = 0.5 × 10^−6^ cm/s) supported the finding of permeability-limited distribution in mPBPK modeling [79]. Another in vitro study [111] showed that PAMPA permeability increased from 0.15 to 0.75 × 10^−6^ cm/s with the increasing pH (i.e., pH ranging from 4.6 to 9.32), which indicates its low permeability over a range of physiologically relevant pH values. In addition to in vitro physicochemical properties, we alternatively applied a typical triexponential function for describing metformin PK (Supplementary Table S5). The total distributional clearance ($CL_{D}$) calculated from these in vivo observations [112,113] (i.e., with initial condition restricted to be $Dose/V_{B}$) was significantly lower than $Q_{CO}$, which also indicates that such modeling reveals that tissue permeability governs the rate of metformin distribution. It is noted that model-dependent CL values from the triexponential fitting were generally comparable with CL values from the separate fitting of mPBPK models in most species, except for dogs. This may be due to the overestimation of AUC in the triexponential function by restricting the initial condition to $Dose/V_{B}$ while the drug was an infused IV over 5 min (as we applied in the mPBPK models). Since $f_{d1}$ (0.0390) is higher than $f_{d2}$ (0.00677) (Table 2), Tissue 1 could be considered as rapidly perfused, while Tissue 2 as slowly perfused, when making a comparison to a traditional three-compartment model.

In this study, the apparent $K_{p}$ values observed for tissues in mouse and rat were collected from the literature as listed in Table 6 (for detailed information, refer to Supplementary Table S3). The $K_{p1}$ values for mouse (0.921) and rat (0.575) obtained from the separate fitting approach appeared to be somewhat underestimated considering the in vivo tissue $K_{p}$ values corresponding to Tissue 1 (e.g., for tissues other than muscle and skin, 0.213–11.3 in mouse and 0.956–4.63 in rat). Nevertheless, the $K_{p2}$ values in the mPBPK modeling for mouse (1.44) and rat (0.466) were consistent with their muscle $K_{p}$ (i.e., 1.03 for mouse and 0.597 for rat, averaged from multiple studies), supporting our kinetic assumption that Tissue 2 included muscle. For the larger species, however, the $K_{p2}$ values were estimated to range from 0.675 to 7.53, which may imply species-dependent metformin partitioning to muscle and skin and/or different tissue components that are kinetically necessary for Tissue 2 in these species. Since metformin is a strong base (pKa = 11.8) and mainly exists in a cationic form in biological matrices, its binding to acidic phospholipids [114] and possible sequestration in the lysosomal/mitochondrial sub-compartments [115,116] may complicate its tissue distribution kinetics with regard to both the extent ($K_{p}$) and rate ($f_{d}$). Interestingly, the metformin distribution within cells showed that concentrations in the mitochondrial fraction (4.6 and 64.5 µM, after 22 h of metformin exposure in Hepa1-6 cells at 75 and 1000 µM) were significantly lower than in the cytosolic fraction (38.2 and 996 µM) [96].

OCT1 is expressed in the basolateral membrane of hepatocytes, which is a major site of pharmacological action for metformin inhibiting glucose production [117]. In the literature [118], the uptake rate of metformin into hepatocytes was highest in monkeys among four species (rats, monkeys, dogs, and man), which is consistent with the highest protein expression of hepatic OCT1 in monkeys [119]. These properties could contribute to the higher estimate of $K_{p1}$ in monkeys (0.615) than rats (0.575) and man (0.484). Other distribution mechanisms may be involved, as $V_{SS}$ is markedly higher in dogs (10.1 L/kg in Table 1 and 6.0 L/kg in Table 5). Similarly, the 4.4-fold higher metformin uptake by mOCT1 than by hOCT1, along with the comparable expression level of the transporter in the liver [120], was consistent with our $K_{p1}$ values for mouse (0.921) and man (0.484). The higher uptake clearance normalized by the mRNA expression levels of hOCT1 compared to rOCT1 (with a factor of 13.1) [121] may explain the difference in our mPBPK model parameter $f_{d1}$ between man (0.993) and rat (0.0694).

Based on the IV PK of metformin reasonably capturing the observations in nine species, the kinetic parameters for absorption could be estimated as shown in Figure 5. There was a decreasing bioavailability of metformin with increasing BW, which was associated with the decrease in its effective absorption rate constant $F\cdot k_{a}$. From a single-pass intestinal perfusion study in rats [33], $P_{eff,rat}$ was lower at a higher concentration of metformin (e.g., 47.1 × 10^−6^ at 10 µg/mL, and 27.0 × 10^−6^ cm/s at 200 µg/mL). The $P_{eff,rat}$ calculated from our mPBPK modeling (4.61 × 10^−6^ cm/s) was much lower than from this in situ study, probably because the 100 mg/kg oral dose given to rats may result in a luminal concentration of 4 mg/mL even under an assumption of instantaneous dissolution of the drug in the total fluid content of the stomach (2.29 g) and small intestine (3.89 g) [122]. This is much higher than 200 µg/mL, showing a probable saturable permeability. In addition, that a saturable oral absorption may be involved is also based on $K_{m}$ values (1.15 to 4 mM) for uptake transporters in the apical membrane of human intestine (e.g., thiamine transporter 2 (THTR-2), plasma membrane monoamine transporter (PMAT), and serotonin reuptake transporter (SERT)) [35]. The differential apical uptake of metformin in the intestinal barrier between hTHTR-2 and mTHTR-2 transporters [123] may be related to the $P_{eff,man}$ (42.6 × 10^−6^ cm/s) being higher than $P_{eff,mouse}$ (7.0 × 10^−6^ cm/s). In addition, the decrease or knockout of OCT1 activity in man and mouse led to the increase of oral absorption of metformin [117]. Since mOCT1/hOCT1 transporters were suggested to play a crucial role in basolateral uptake of metformin into intestinal tissue [124], higher $P_{eff,man}$ may be involved with the species-dependent transport of metformin by intestinal OCT1. From our mPBPK modeling, the $P_{eff,in\;vivo}$ was estimated to be relatively higher in minipig, dog, and human than other species (Figure 5). Considering the probable absence of solubility limitations of metformin in gut lumen, species differences in $P_{eff,in\;vivo}$ may result, at least in part, from the water content per gut length being higher in pig, dog, and human [122], leading to enhanced active uptake across the gut wall with lower effective concentrations in the lumen. The presence of THTR-2 and OCT1 transporters in the GI tract undoubtedly account for the high tissue-to-plasma ratios observed in mice and rats (Table 6).

Proctor et al. [34] examined metformin transport in Caco-2 cells to demonstrate carrier-mediated apical uptake that was seven-fold greater than basolateral efflux. The latter is limiting for transcellular transport and appears to account for the high metformin $K_{p}$ values in GI tissues (Table 6). The $P_{eff,man}$ predicted from this in vitro Caco-2 permeability (e.g., $\log P_{eff}=0.4926\times \log P_{app}-0.1454$, where $P_{app}$ = 500 nm/s for metformin) [125] was 15.3 µm/s, which is comparable to $P_{eff,man}$ from our modeling analysis (42.6 µm/s). Based on the mPBPK model, metformin exhibits partial bioavailability in man (F= 0.485), compared to the fraction absorbed from the enteric compartment ($F_{a}=$ 0.185) calculated from a physiological absorption model (e.g., $F_{a}=1-{\left(1+0.54P_{eff}\right)}^{-7}$, using the $P_{eff}$ of 15.3 µm/s in the Advanced Compartmental Absorption and Transit (ACAT) model) [126]. Using $T_{SI}$ and R (gut radius) values collected from the literature (Supplementary Table S2), the coefficient corresponding to $P_{eff}$ term (i.e., $2T_{SI}/7R$) generally decreased with increasing body size. Thus, if there is no species difference in $P_{eff}$, which can be assumed to be the same with $P_{eff,rat}$ (e.g., 27.0 × 10^−6^ cm/s) or $P_{eff,man}$ predicted from Caco-2 cell permeability (e.g., 15.3 × 10^−6^ cm/s), the ACAT model consisting of seven enteric compartments argues for a decreasing F with increasing BW (Supplementary Figure S2). This assessment suggests that the joint role of metformin permeability and gut physiology accounts for the decreasing bioavailability with increasing BW in these species. Species differences in bioavailability were judged to be considerable for a variety of compounds in a literature review [127]. However, the general applicability of this conclusion for $F_{a}$ needs further evaluation by factoring in the first-pass hepatic and gut wall extraction from the overall oral bioavailability.

Despite the abundant evidence for the involvement of carrier-mediated transport, the assumption of linear PK appeared to be adequate for describing the plasma PK of metformin in the current meta-analysis. In single-dose clinical studies [37,40], the terminal half-life of metformin based on urinary excretion rates was 4.22 to 5.11 times longer than that in plasma. However, this appears due to the longer sampling time in urine, which may better capture a late terminal phase. Metformin has a reported longer PK half-life when followed in erythrocytes (23.4 h) compared to plasma (2.7 h) after single doses [39]. This nonequilibrium between plasma and erythrocytes [37] is ascribed to a slow uptake (t_1/2_ > 80 h) and slow efflux (t_1/2_ > 32 h) from the cells [128]. While the PK profiles in these publications may not have been monitored over the same or for a sufficient period of time, Kajbaf et al. [129] followed metformin washout kinetics in patients with regard to both plasma and erythrocytes over 5 to 14 days. Although these patients had clinical complications, such as acute renal failure, the corresponding half-lives were 51.9 and 43.4 h. It is likely that many single-dose studies failed to assess metformin disposition over sufficiently long times to account for late phases and small amounts of the retained drug.

The marked difference between $V_{SS}$ (6–10 L/kg for dogs and 0.432–0.856 L/kg for man) and $V_{d,area}$ (44.8 L/kg for dogs and 0.859–3.83 L/kg for man) [36,37,40,71] is likely related to differences in transporters and related tissue uptake. From the collected literature information (Supplementary Table S3), tissue $K_{p}$ values at early times (e.g., <24 h) ranged 0.359 to 0.771 for muscle and 0.0354 for brain in mouse [55,130,131], while the prolonged treatment of metformin (i.e., from 7 to 30 days) produced $K_{p}$ values of 1.00–2.06 for muscle and 0.174–0.257 for the brain [132]. Similarly in rats, the brain $K_{p}$ increased from 0.2 (at 1 h) to 1.48 (at 24 h) [133], while the $K_{p}$ values for muscle were 0.455 at 2 h of day 1 and 0.738 at 2 h of day 7 [134]. This time-dependent increase in tissue uptake of metformin may be related to differing terminal phase half-lives between the plasma and erythrocytes/tissues or may reflect drug-induced changes in the distribution. Collectively, therefore, carrier-mediated transport and/or continuous tissue redistribution may vary within or among species and alter the parameters in the PBPK model. More extensive studies that follow plasma, tissue, and urinary excretion of metformin during multiple dosing and washout are needed.

An insightful assessment of metformin PK in dogs was carried out by Wesolowski et al. [71]. Their fitting algorithm accounts for the early distribution kinetics during and after the IV infusion (much like seen in PET scans by Gormsen et al. [135]) and handles the late disposition phase as a power function that captures the straight line phase on a log–log plot. This method applies a gamma-Pareto convolution that predicts slow continuing distribution consistent with the slow kinetics associated with red blood cells, the very low P-values (Table 4), and the small $f_{d}$ values (Table 2 and Table 5) for metformin. This approach yields a lower CL than the NCA analysis, and their mean CL was 15.1 compared to our mPBPK value of 19.3 mL/min/kg. It is also likely that $V_{SS}$ values are underestimated when applying an exponential to a power function curve. While this elegant method may better capture the terminal phase of metformin PK in dogs, a species with unusually high $V_{SS}$ (Table 1), it appears to be only applicable to single PK profiles and cannot, as yet, be utilized for tandem (oral and IV) PK profiles, as assessed here.

The present meta-assessment utilized the mPBPK modeling approach to compare the PK properties of metformin across nine species and supported the distribution model parameters with established calculation methods [81,82] in Table 4 and extensive tissue-to-plasma ratios for rodents summarized from the literature (Table 6). Typically, full PBPK models are built using both types of information. Burt et al. [109] utilized the Simcyp Simulator (Simcyp Ltd., Certara Co., Sheffield, UK) to predict metformin plasma concentrations in man, assuming F= 0.7, calculated $K_{p}$ [114] values for various tissues, the $k_{a}$ re-estimated using oral dose PK data, and renal and hepatic uptake related to transporter function with optimization using observed plasma and urinary excretion data. With such manipulations, single oral dose profiles of metformin were reasonably ‘predicted’. Hanke et al. [42] carried out a similar PBPK modeling effort using PK-Sim^®^ (Bayer Technology Services, Leverkusen, Germany) to capture the metformin profiles from 22 studies with doses ranging 0.001 to 2550 mg for up to 696 h during multiple dosing. The analysis included $K_{p}$ values based on PET data obtained in plasma, blood, kidney, liver, intestines, and muscle over 1.5 h by Gormsen et al. [135]. Most of the assessment was based on matching plasma concentration profiles, which did not lend insight into longer time tissue distribution/accumulation kinetics of metformin.

The present meta-assessment has some limitations. While we sought all available PK studies of metformin that could be found in PubMed and by reference tracking, the drug has been subject to immense scrutiny, and undoubtedly, some relevant material may be missed. Diverse analytical methods ranging from early HPLC-UV to more recent LC-MS/MS may have a range of specificities and sensitivities (Table 1). Most of the data were digitized and dependent on published graphs with limitations based on single doses and in the duration of sampling (Figure 3 and Figure 4). While metformin is purported to have linear kinetics (as we assumed), this depends on substrate exposures being lower than transporter $K_{m}$ values. The washout PK in some species (like dogs) may better follow a power function, indicating complexities in distribution mechanisms that need tissue PK data and longer-term studies for better resolution. Nevertheless, the mPBPK models represent a state-of-the-art approach to modeling, summarizing, and comparing available single-dose profiles of metformin in the various species. Extending this modeling approach to include and compare oral and IV profiles across species provided unique insights into the absorption properties of metformin.

Upon completion of our analysis, Morse et al. [136] published a report demonstrating the interspecies comparison of the expression and activities of OCT transporters in the liver, which was investigated in relation to metformin PK. Using a single IV dose of 5-mg/kg metformin, wild-type rats showed comparable PK properties with the datasets (50, 100, and 200 mg/kg) used in the current analysis [52,53], in terms of CL (29.4 versus 23.6 to 26.4 mL/min/kg) and $CL_{R}$ (i.e., 21.8 versus 17.8 to 19.5 mL/min/kg). Consistent with the previous PBPK model suggesting the importance of apical efflux process of metformin by MATE1/2 transporters [109,110], single knockouts of the rOCT1 or rOCT2 transporters did not affect the plasma PK and tissue partitioning of metformin in rats. Different from man and mouse [117], however, knockout of the rOCT1 transporter exhibited no significant effect on the oral absorption of metformin in rats. It is noted that their measurements of metformin concentrations in plasma up to 24 h after IV administrations led to observing a longer half-life (5.12 h) compared to 2.1–2.5 h determined using the data for 10 h [52]. Thus, mean residence time in the body increased by using the longer half-life values resulted in higher $V_{SS}$ (2.25 L/kg). The tissue-to-plasma ratios observed for various tissues at 4 h after the IV dose may govern the determination of $V_{d,area}$ rather than $V_{SS}$ [137]. Tissue partitioning properties mostly in a range far above the $V_{SS}$ value in the literature (2.25 L/kg) or in our current analysis (0.536 L/kg) may imply the involvement of low tissue permeability (Table 2 and Table 5) and/or slow/continuous redistribution of metformin between plasma and tissues in rats, as discussed above.

In conclusion, we collected PK profiles from nine species for metformin, an effective antidiabetic agent widely used for various clinical and veterinary purposes. Utilizing a mPBPK model, the common features of metformin distribution that appear conserved among species were assessed in the joint fitting approach, while potential differences in PK parameters were evaluated in the individual fittings, with the results compared with the related literature data. Despite the lack of integrated information for tissue PK of metformin, a priori in silico calculations of physicochemical properties adopted in mPBPK models enabled the physiological interpretation of the absorption and disposition PK of metformin in various species. Unique perspectives were provided regarding the bioavailability that was found to be reduced with increased BW. This study provided a systematic review of the literature datasets and a quantitative analysis of metformin PK in all the available species and demonstrated the advantages of the simultaneous fitting of data from multiple species using system-specific physiological and anatomical variables. Further experimental studies are needed to resolve the mysteries of metformin absorption and distribution with the implementation of full and extended PBPK models utilizing longer term data to characterize its kinetics more completely.

## 4. Methods

### 4.1. Data Collection and Basic Allometric Scaling

The PK parameters for metformin including the systemic clearance (CL), oral clearance (CL/F), renal clearance ($CL_{R}$), and steady-state volume of distribution ($V_{SS}$) were collected from the literature for nine species (Table 1) based on a PubMed search and reference tracking. If not directly obtainable in the literature, these PK parameters were either calculated from available descriptors or obtained by noncompartmental analysis (NCA) of the plasma concentration–time profiles that were digitized from the published graphs using GetData Graph Digitizer version 2.26 (http://getdata-graph-digitizer.com/, accessed on 1 May 2021).

The physiological and anatomical variables necessary for mPBPK modeling were obtained for the species in which the metformin PK was found in the literature (mouse, rat, rabbit, cat, monkey, minipig, dog, man, and horse). The blood volumes were from one literature source [138], except for man [139]. The cardiac output for each species was determined by an allometric relationship [66]:

(1) $$Q_{CO}=0.235\cdot BW^{0.75}$$

In addition, the renal plasma flow (RPF) [65,66,67,68,69,70,71,72], glomerular filtration rate (GFR) [70,105,140,141], gut radius (R) [70,142,143,144,145,146], luminal volume of the GI tract ($V_{Lumen}$ for the stomach and small intestine) [122], and the small intestinal transit time ($T_{SI}$) [122,147,148,149,150,151] were obtained for each species. The detailed values including the anatomical volumes and blood perfusion rates to muscle ($V_{MU}$, $Q_{MU}$) and skin ($V_{SK}$, $Q_{SK}$) for the species-specific input variables are summarized in Supplementary Table S2. Allometric relationships of these variables among the 9 species are provided in Supplementary Figure S1.

### 4.2. mPBPK Modeling via Joint Fittings

In the mPBPK model structure (Figure 2), blood and two peripheral tissue compartments (Tissues 1 and 2) were assumed. The differential equations used for the joint fitting are:

(2) $$V_{B}R_{b}\frac{dC_{p}}{dt}=Input+Q_{CO}\cdot f_{d1}\cdot R_{b}\cdot \left(\frac{C_{1}}{K_{p}}-C_{p}\right)+Q_{CO}\cdot f_{d2}\cdot R_{b}\cdot \left(\frac{C_{2}}{K_{p}}-C_{p}\right)-CL\cdot C_{p}$$

(3) $$V_{1}\frac{dC_{1}}{dt}=Q_{CO}\cdot f_{d1}\cdot R_{b}\cdot \left(C_{p}-\frac{C_{1}}{K_{p}}\right)$$

(4) $$V_{2}\frac{dC_{2}}{dt}=Q_{CO}\cdot f_{d2}\cdot R_{b}\cdot \left(C_{p}-\frac{C_{2}}{K_{p}}\right)$$

where $C_{p}$ is the plasma concentration of metformin, $C_{1}$ and $C_{2}$ are the metformin concentrations in Tissues 1 and 2, $f_{d1}$ and $f_{d2}$ are the fractional distribution parameters for Tissues 1 and 2, $V_{1}$ and $V_{2}$ are the anatomical volumes of Tissues 1 and 2, $Q_{CO}$ is cardiac output, $K_{p}$ is the tissue-to-plasma partition coefficient, $R_{b}$ is the blood-to-plasma ratio, $V_{B}$ is the blood volume, and CL is the systemic clearance. The IV Input was defined in accordance with the methodology of the literature source (e.g., bolus or short infusion, where Input= Dose/Duration of infusion). The initial conditions for $C_{1}$ and $C_{2}$ were set as zero. The blood partitioning properties of metformin (e.g., $R_{b}$ and $f_{up}$ (free fraction in the plasma)) were found to be in unity [39,109,128].

The BW (in kg) for each species was obtained from the publications with the metformin PK data (Table 1). The tissue volume fractions for Tissue 1 ($f_{t}$) and Tissue 2 ($1-f_{t}$) were used to calculate $V_{1}$ and $V_{2}$, assuming the density of all tissues to be in unity:

(5) $$V_{1}=\left(BW-V_{B}\right)\cdot f_{t}$$

(6) $$V_{2}=\left(BW-V_{B}\right)\cdot \left(1-f_{t}\right)$$

Due to the low permeability characteristics of metformin (e.g., in vitro PAMPA permeability of 0.5 × 10^−6^ cm/s) [74], the permeability-limited distribution [79] was assumed by using $f_{d,total}$ conserved across all species:

(7) $$f_{d,total}=f_{d1}+f_{d2}\leq 1$$

The primary estimated parameters, along with CL, were $K_{p}$, $f_{t}$, $f_{d,total}$, and $f_{d1}$, while $f_{d2}$ was a secondary parameter. It can be noted that, except for CL, the other parameters are dimensionless.

### 4.3. mPBPK Modeling via Separate Fittings

Based on the collected PK data (Table 1), species differences were observed in metformin PK (e.g., bioavailability from 7.1% to 100% and $V_{SS}$ from 0.321 to 10.1 L/kg). Since the involvement of various transporters is evident for metformin PK [35], there is a possibility that species-dependent transport activity may lead to different PK variables for each species. Therefore, we further conducted the mPBPK model fitting to oral and IV profiles for each species separately with the addition of more PK data.

A priori in silico calculation of the tissue distribution parameters ($K_{p}$) was carried out for 11 typical tissues of rats as applied in whole-body PBPK (WB-PBPK) models. Physicochemical (e.g., log P = −1.43, pKa = 11.8) [30,32] and blood partitioning properties of metformin ($f_{up}$ = 1, $R_{b}$ = 1) [39,109,128] were used. The $K_{p}$ values from two calculation methods (i.e., Poulin and Theil [81], and Berezhkovskiy [82]) were applied for the prediction of $V_{SS}$ amongst the four methods found in the GastroPlus PBPK simulator (version 9.8.0002; Simulations Plus, Inc., Lancaster, CA, USA). For the calculation of $f_{d}$, two approaches were considered for the tissue permeability coefficient P: (i) in vitro PAMPA P (0.5 × 10^−6^ cm/s) [74] and (ii) the P-value fitted to the rat PK data (0.0102 × 10^−6^ cm/s). For the latter case, a WB-PBPK model structure employing $S_{eff}$ (i.e., the effective surface area across the interface between the systemic circulation and tissues) [79] was used. For the calculation of $f_{d,tissue}$:

(8) $$f_{d,tissue}=1-e^{-\frac{f_{up}S_{eff}P}{Q_{T}R_{b}}}$$

where $Q_{T}$ is the blood flow to each tissue. Mean transit time (MTT) through the tissues was calculated as:

(9) $$MTT=\frac{V_{T}K_{p}/R_{b}}{Q_{T}f_{d,tissue}}$$

where $V_{T}$ is the anatomical tissue volume.

Compared to traditional compartment models, mPBPK models enable a more physiological interpretation of the systemic PK. Since $f_{t}$ determined by the joint fitting was 0.172 with a single $K_{p}$ assumption (see Results), it was reasoned that muscle, the anatomically largest organ, is included in Tissue 2 (e.g., $V_{MU}/\left(BW-V_{B}\right)$ ranges from 0.411 to 0.574 (>0.172) in the 9 species; Supplementary Table S2). Since our in silico calculations suggested skin as the kinetically largest tissue for variable P (see Results), then Tissue 2, the slowly equilibrating compartment, also included skin (i.e., calculated $f_{t}$ value ranging from 0.472 to 0.678 for Tissue 2 in the 9 species). The differential equations used for the separate fittings are:

(10) $$V_{B}R_{b}\frac{dC_{p}}{dt}=Input+Q_{1}\cdot f_{d1}\cdot R_{b}\cdot \left(\frac{C_{1}}{K_{p1}}-C_{p}\right)+Q_{2}\cdot f_{d2}\cdot R_{b}\cdot \left(\frac{C_{2}}{K_{p2}}-C_{p}\right)-CL\cdot C_{p}$$

(11) $$V_{1}\frac{dC_{1}}{dt}=Q_{1}\cdot f_{d1}\cdot R_{b}\cdot \left(C_{p}-\frac{C_{1}}{K_{p1}}\right)$$

(12) $$V_{2}\frac{dC_{2}}{dt}=Q_{2}\cdot f_{d2}\cdot R_{b}\cdot \left(C_{p}-\frac{C_{2}}{K_{p2}}\right)$$

where $Q_{2}$ is the blood flow rate to Tissue 2 ($Q_{MU}+Q_{SK})$, $Q_{1}$ is the blood flow rate to Tissue 1 ($Q_{CO}-Q_{2}$), $V_{2}$ is the anatomical volume of Tissue 2 ($V_{MU}+V_{SK}$), $V_{1}$ is the anatomical volume of Tissue 1 ($BW-V_{B}-V_{2}$), and $K_{p1}$ and $K_{p2}$ are the tissue-to-plasma partition coefficients for Tissues 1 and 2. The IV doses had bolus or infusion inputs, while, for the oral administration, the drug Input from the absorption compartment is:

(13) $$\frac{dX_{a}}{dt}=-k_{a}X_{a}$$

(14) $$Input=F\cdot k_{a}\cdot X_{a}$$

where $X_{a}$ is the drug amount at the absorption site with the initial condition of $X_{a}\left(0\right)=Dose$, $k_{a}$ is the first-order absorption rate constant, and F is the bioavailability.

### 4.4. Model Fittings

For the nonlinear regression analysis, the maximum likelihood method in ADAPT 5 was used [152]. The variance model was:

(15) $$V_{i}={\left(\sigma_{1}+\sigma_{2}Y_{i}\right)}^{2}$$

where $V_{i}$ is the variance of the *i*th data point, $Y_{i}$ is the *i*th model prediction, $\sigma_{1}$ and $\sigma_{2}$ are the variance model parameters that were estimated together with the system parameters during the model fittings. The goodness-of-fit of the model was monitored by visual inspection, Akaike Information Criterion (AIC), and Coefficient of Variation (CV%) of the estimates. The ADAPT model code for the joint mPBPK is provided in the Supplementary Materials. The NCA were conducted using WinNonlin Professional 5.0.1. software (Pharsight Corporation, Mountain View, CA, USA).

## Figures and Tables

**Table 1:** Literature reports and pharmacokinetic parameters of metformin after intravenous and oral doses in various species.

Reference	Species	Strain	Sex	Body Weight (kg)	Dosing Route	Dose (mg/kg)	Assay	CL (mL/min/kg)	CL/F (mL/min/kg)	$CL_{R}\;(\mathrm{mL}/\min/\mathrm{kg})$	RPF (mL/min/kg)	$V_{SS}\;(\mathrm{mL}/\mathrm{kg})$
Higgins et al. (2012) [54]	Mouse	FVB	M	-	IV *	5	LC-MS/MS	81.7		40.0	60 ^a^	1840
					PO *	10	LC-MS/MS		138			
Nakamichi et al. (2013) [55]	Mouse	C57BL/6J	M	-	IV Infusion	0.12 mg/kg/min	HPLC-UV	60.7		60.3		
					PO *	50, 175	HPLC-UV		77.5, 59.8 **			
Choi et al. (2006) [52]	Rat	Sprague-Dawley	M	0.20–0.31	IV *	50, 100, 200	HPLC-UV	23.6–26.4		17.8–19.5	24.8 ^b^	586–693
					PO	50, 100, 200	HPLC-UV		76.0–82.0	37.4–39.6		
Choi et al. (2010) [53]	Rat	Sprague-Dawley	M	0.19–0.30	IV	100	HPLC-UV	14.7		11.6		383
					PO *	100	HPLC-UV		34.1	30.9		
Bouriche et al. (2020) [56]	Rabbit	New Zealand	F	-	IV *	5	HPLC-UV	2.05			13.1 ^c^	321
					PO *	5	HPLC-UV		5.67			
Choi and Choi (2003) [57]	Rabbit	New Zealand	M	2.0–2.2	PO *	15	HPLC-UV		6.01			
Michels et al. (1999) [58]	Cat	Domestic shorthair	-	4.3–6.5	IV *	25	HPLC-UV	2.5		2.17	13.8 ^d^	550
					PO *	25	HPLC-UV		5.21			
Nelson et al. (2004) [59]	Cat	-	M	4–5	PO *	50 mg	HPLC-UV		9.05			
Shen et al. (2016) [60]	Monkey	Cynomolgus	M	5–7	IV *	3.9	LC-MS/MS	11.2		10.7	9.61^e^	802 **
Heinig and Bucheli (2004) [61]	Monkey	Cynomolgus	-	-	PO *	250	LC-MS/MS		93.5 **			
Patel et al. (2017) [62]	Minipig	Hanford	M	14.3	IV *	0.5	LC-MS/MS	9.7			9.36 ^f^	2260 **
					PO *	5	LC-MS/MS		14.8 **			
Johnston et al. (2017) [51]	Dog	Mixed	-	25.7–29.2	IV *	24.8	FIA-MS/MS	24.1			17.9 ^g^	10100
					PO *	19.1	FIA-MS/MS		77.7			
Sirtori et al. (1978) [36]	Man	Healthy	4 M 1 F	64–81	IV *	926 mg	GC-MS	6.13		4.65	7.81 ^b^	432 **
Pentikäinen et al. (1979) [40]	Man	Healthy	1 M 2 F	58–63	IV *	500 mg	LSC	7.61		7.52		856 **
			2 M 3 F	56–80	PO *	500 mg	LSC		16.3	7.06		
Tucker et al. (1981) [37]	Man	Healthy	4 M	64–83	IV *	250 mg	GC-EC	10.1		7.83		511 **
					PO *	500, 1500 mg	GC-EC		18.9, 21.5	7.42, 7.16		
Lee and Kwon (2004) [63]	Man	Healthy	22 M	55–89	PO *	500 mg	HPLC-UV		16.2			
Hustace et al. (2009) [64]	Horse	-	-	531.8	IV *	6 g	HPLC-UV	10.8			12.1 ^h^	2250 **
					PO *	6 g	HPLC-UV		152			

* Dataset included in the modeling analysis. ** Calculated from the digitized data. ^a^ Thuesen et al. (2014) [65]. ^b^ Brown et al. (1997) [66]. ^c^ Sweeny et al. (2009) [67]. ^d^ Lindstedt and Schaeffer (2002) [68]. ^e^ Values adopted in Simcyp V19 [69]. ^f^ Suenderhauf and Parrott (2013) [70]. ^g^ Wesolowski et al. (2019) [71]. ^h^ Holdstock et al. (1998) [72].

**Table 2:** Pharmacokinetic parameters of metformin jointly fitted to the minimal physiologically based pharmacokinetic model across 9 species^a,b^.

	Definition	Estimate (CV%)
$K_{p}$	Tissue-to-plasma partition coefficient	1.29 (9.67)
$f_{d,total}$	Total sum of fractional distribution parameters ($f_{d1}+f_{d2}$)	0.0457 (8.12)
$f_{d1}$	Fractional distribution parameter for Tissue 1	0.0390 (8.91)
$f_{d2}$	Fractional distribution parameter for Tissue 2	0.00677 (7.59) ^c^
$f_{t}$	Fraction of total tissue volume for Tissue 1	0.172 (8.87)

^a^ The jointly-fitted CL values are listed in Table 3. ^b^ AIC = 248.19. ^c^ Estimated as a secondary parameter by $f_{d2}=f_{d,total}-f_{d1}$.

**Table 3:** Comparison of systemic clearance values of metformin for 9 species based on the indicated methods of calculation/estimation (CV% obtained by fitting).

Species (Body Weight, kg)	Reported CL (mL/min/kg) *	NCA CL (mL/min/kg)	mPBPK Joint CL (mL/min/kg)	mPBPK Individual CL (mL/min/kg)	RPF (mL/min/kg)
Mouse (0.025)	81.7	81.8	32.9 (6.05)	65.8 (26.9)	60
Rat (0.25)	23.6–26.4	20.8–21.3	23.5 (3.80)	23.2 (4.00)	24.8
Rabbit (2)	2.05	2.05	1.43 (14.5)	1.30 (38.2)	13.1
Cat (5)	2.5	2.55	2.90 (4.78)	2.78 (7.11)	13.8
Monkey (7)	11.2	11.0	12.5 (5.87)	15.1 (7.71)	9.61
Minipig (14)	9.7	9.18	6.08 (6.37)	9.32 (9.72)	9.36
Dog (28)	24.1	20.2	9.01 (4.15)	19.3 (15.8)	17.9
Man (70)	6.13–10.1	6.19–9.47	5.20 (3.77)	7.74 (4.19)	7.81
Horse (530)	10.8	8.25	5.51 (5.49)	6.89 (11.0)	12.1

* References are provided in Table 1.

**Table 4:** A priori in silico calculation of the tissue distribution parameters ($K_{p}$ and $f_{d}$) for 11 typical tissues of rats in the WB-PBPK. The $K_{p}$ values were predicted using the Poulin and Theil [81] and Berezhkovskiy [82] methods built in the GastroPlus PBPK simulator, except for the gut. In vitro PAMPA P (0.5 × 10^−6^ cm/s) and fitted P (0.0102 × 10^−6^ cm/s) were used to calculate the mean transit time (MTT).

Tissue ^a^	*K_p_*	In Vitro PAMPA *P*		Tissue ^a^	*K_p_*	Fitted P to In Vivo Profile	
		$f_{d,tissue}$	MTT (min)			$f_{d,tissue}$	MTT (min)
Kidney	0.82	0.88	0.176	Kidney	0.82	0.0423	3.66
Lung	0.83	0.0648	0.199	Heart	0.82	0.00137	7.54
Heart	0.82	0.832	0.324	Gut ^b^	0.81	0.0357	8.53
Liver	0.76	0.86	0.391	Liver	0.76	0.0393	8.56
Adipose	0.16	0.959	0.589	Adipose	0.16	0.0632	8.94
Gut ^b^	0.81	0.975	0.636	Lung	0.83	0.0727	9.42
Spleen	0.81	0.588	0.893	Muscle	0.79	0.0179	25.1
Bone ^c^	0.47	0.744	1.23	Spleen	0.81	0.0274	29.3
Brain	0.86	0.425	2.24	Bone ^c^	0.47	0.0113	33.3
Muscle	0.79	1	4.84	Brain	0.86	0.1930	84.6
Skin	0.69	0.913	7.30	Skin	0.69	0.0486	137

^a^ Tissues were arranged in ascending order of MTT for each case. ^b^ $K_{p}$ value predicted by Method 1 in Simcyp V19 (Berezhkovskiy [82]).; ^c^ $K_{p}$ value for red marrow predicted by GastroPlus 9.8.

**Table 5:** Pharmacokinetic parameters of metformin separately fitted to the minimal physiologically based pharmacokinetic model across 9 species.

	Estimate (CV%)								
	Mouse	Rat	Rabbit	Cat	Monkey	Minipig	Dog	Man	Horse
$K_{p1}$	0.921 (81.0)	0.575 (10.2)	0.479 (19.9)	0.405 (20.6)	0.615 (21.2)	0.875 (20.5)	4.31 (38.1)	0.484 (13.3)	0.175 (27.4)
$K_{p2}$	1.44 (50.0)	0.466 (9.62)	2.15 (93.5)	2.69 (18.6)	3.27 (17.5)	4.13 (20.8)	7.53 (30.2)	0.669 (11.9)	0.884 (213)
$f_{d1}$	0.379 (163)	0.0694 (14.5)	0.106 (36.2)	0.0789 (38.5)	0.158 (32.3)	0.966 (34.9)	0.765 (83.6)	0.993 (44.4)	0.106 (46.3)
$f_{d2}$	0.239 (69.5)	0.0157 (11.7)	0.0184 (29.0)	0.0321 (14.3)	0.0482 (21.9)	0.0472 (22.7)	0.130 (30.8)	0.171 (22.3)	0.0455 (81.1)
CL (mL/min) ^a^	1.65 (26.9)	5.81 (4.00)	2.60 (38.2)	13.9 (7.11)	106 (7.71)	131 (9.72)	540 (15.8)	542 (4.19)	3650 (11.0)
F	0.995 (27.5)	0.675 (7.28)	0.334 (11.0)	0.501 (8.63)	0.157 (10.3)	0.474 (13.6)	0.203 (17.2)	0.485 (5.54)	0.0873 (11.7)
$k_{a}$ (h^−1^)	0.375 (15.4)	0.246 (4.05)	0.424 (16.0)	0.402 (11.1)	0.209 (9.71)	0.390 (15.7)	0.390 (13.0)	0.253 (5.42)	0.144 (11.7)
$V_{SS}$ (L/kg) ^b^	1.21 (51.5)	0.536 (7.48)	1.49 (79.8)	1.56 (16.9)	1.89 (16.3)	2.65 (18.8)	6.00 (30.5)	0.603 (7.36)	0.574 (156)

^a^ Fitted CL values are listed in Table 3. ^b^ $V_{SS}$ calculated as a secondary parameter ($V_{SS}=\left(V_{B}+V_{1}\cdot K_{p1}+V_{2}\cdot K_{p2}\right)/BW$).

**Table 6:** Summary of measured tissue and blood partition coefficients in four species *.

Species	Tissue	.	Mean (Range) **
		No. of values	$K_{p}$
Mouse	Liver	16	3.47 (1.72–7.10)
	Brain	5	0.213 (0.0354–0.257)
	Kidney	12	8.74 (3.35–20.5)
	Muscle	8	1.03 (0.359–2.06)
	Heart	3	0.610 (0.519–0.712)
	Adipose	1	0.471
	Stomach	4	6.38 (4.67–9.03)
	Small intestine	6	11.3 (0.837–21.1)
	Colon	4	7.72 (4.52–13.9)
	Salivary gland	2	3.03 (2.60–3.45)
Rat	Liver	10	3.07 (0.368–6.83)
	Brain	5	0.8 (0.2–1.48)
	Kidney	9	4.04 (0.128–5.92)
	Muscle	2	0.597 (0.455–0.738)
	Spleen	1	0.956
	Gut	1	4.63
		No. of values	$R_{b}$
Rat	Blood	2	1.18 (0.98–1.37)
Man	Blood	2	1.03 (0.83–1.23)
	(free fraction in the)	No. of values	$f_{u}$
Rat	Plasma	3	0.873 (0.849–0.897)
Dog	Plasma	3	0.904 (0.83–0.951)
	Blood	1	0.920
Man	Plasma	6	0.938 (0.75–1.0)
	Blood	1	0.932

* Detailed literature information is listed in Supplementary Table S3. Data from normal (nondiabetic) species were included after an outlier test based on the interquartile range rule. ** For data presented as a range from a single study, the average value of the maximum and minimum was adopted.

## Data Availability

All the data generated during this study are included in this article. Further datasets digitized and/or analyzed during the study are available from the corresponding author upon reasonable request.

## Supplementary Materials

The following are available online at https://www.mdpi.com/article/10.3390/ph14060545/s1: Figure S1: Physiological and anatomical information (i.e., $V_{SK}$, $V_{MU}$, $Q_{SK}$, $Q_{MU}$, RPF, and GFR) collected from different sources (Supplementary Table S2) showed an allometric relationship among 9 species (i.e., mouse (0.025 kg), rat (0.25 kg), rabbit (2 kg), cat (5 kg), monkey (7 kg), minipig (14 kg), dog (28 kg), man (70 kg), and horse (530 kg))., Figure S2: ACAT model-based estimation of bioavailability using $F_{a}=1-{\left(1+2P_{eff}T_{SI}/7R\right)}^{-7}$ (7-enteric compartments), assuming that the effective intestinal permeability ($P_{eff}$) of the 9 species is the same with that of rat ($P_{eff,rat}$), and that of man ($P_{eff,man}$) predicted from Caco-2 cell permeability, which were compared with bioavailability determined from our mPBPK modeling (1-enteric compartment as a cylinder). Table S1: Literature reports and pharmacokinetic parameters of metformin given intravenously in different species. Table S2: Summary of the physiological input variables for the meta-analysis of metformin pharmacokinetics in various species. Table S3: Literature information collected for tissue distribution and blood partitioning of metformin. Table S4: Calculation of the steady-state volume of distribution ($V_{SS}$) using in vivo tissue $K_{p}$ values based on the equation $V_{SS}=V_{B}+\sum V_{T,i}K_{p,i}$ [84]. Tissue $K_{p}$ values were assumed to be muscle $K_{p}$ (1.03 for mouse and 0.597 for rat) if unavailable in Table 6. Table S5: Model fitting results using a triexponential function ($C_{p}\left(t\right)=C_{1}e^{-\lambda_{1}t}+C_{2}e^{-\lambda_{2}t}+C_{3}e^{-\lambda_{3}t}$) for metformin PK in various species (CV% obtained by fitting). It is noted that five parameters ($C_{1}$, $\lambda_{1}$, $C_{2}$, $\lambda_{2}$, and $\lambda_{3}$) were optimized, while $C_{3}$ values were estimated as a secondary parameter ($C_{3}=C_{0}-C_{1}-C_{2}$; $C_{0}=Dose/V_{B}$). In addition, the ADAPT model code for the joint mPBPK is provided in the Supplementary Materials.
