# Supplementary material for: Meta-Assessment of Metformin Absorption and Disposition Pharmacokinetics in Nine Species
Source: Pharmaceuticals (Basel). 2021 Jun 7;14(6):545. doi: 10.3390/ph14060545 (PMC8226464; doi:10.3390/ph14060545)
Supplement: Supplementary file 1 [file pharmaceuticals-14-00545-s001.zip › Supplementary_material.pdf]

Supplementary Table S1. Literature reports and pharmacokinetic parameters of metformin given intravenously in different species

Reference	Species	Strain	Sex	Dosing route	Dose (mg/kg)	Assay	CL (mL/min/kg)	CL_R (mL/min/kg)	V_{SS} (mL/kg)
Tsuda et al (2009) [26]	Mouse	C57BL/6	M	IV	5	HPLC-UV	40.7	37.2	994
Higgins et al (2012) [27]*	Mouse	FVB	M	IV	5	LC-MS/MS	81.7	40.0	1840
Nakamichi et al (2013) [28]	Mouse	C57BL/6J	M	IV(+IF)	6 (+0.12 mg/min/kg)	HPLC-UV	60.7	60.3	-
Chen et al (2015) [29]	Mouse	C57BL/6J	M	IV	50	LSC	18.6	-	1480
Shirasaka et al (2016) [30]	Mouse	FVB	M	IV	8	LSC	12.7	-	1740
Kakemi et al (1983) [31]	Rat	Wistar	M	IV	50	GC-MS	42.8**	-	1920**
					100	GC-MS	14.5**	-	1020**
					200	GC-MS	7.73**	-	880**
Choi et al (2006) [32]*	Rat	Sprague-Dawley	M	IV	50	HPLC-UV	26.4	19.5	693
					100	HPLC-UV	24.5	18.7	586
					200	HPLC-UV	23.6	17.8	586
Choi and Lee (2006) [33]	Rat	Sprague-Dawley	M	IV	100	HPLC-UV	17.2-23.0	10.5-13.4	566-844
Choi et al (2007) [34]	Rat	Sprague-Dawley	M	IV	100	HPLC-UV	20.2	11.7	797
Choi et al (2007) [35]	Rat	Sprague-Dawley	M	IV	100	HPLC-UV	21.7	13.6	444
Choi et al (2007) [36]	Rat	Sprague-Dawley	M	IV	30 μmol/mL/kg	HPLC-UV	19.0	-	426

Maeda et al (2007) [37]	Rat	Wistar	M	IV	1	LSC	12.3	-	764
Lee et al (2008) [38]	Rat	Sprague-Dawley	M	IV	100	HPLC-UV	19.6	11.7	655
Choi et al (2008) [39]	Rat	Sprague-Dawley	M	IV	100	HPLC-UV	20.3-22.6	11.7-12.4	651-826
Choi et al (2008) [40]	Rat	Sprague-Dawley	M	IV	100	HPLC-UV	16.8	9.46	539
Jin et al (2008) [41]	Rat	Sprague-Dawley	M	IV	5	HPLC-UV	21.4	17.6	766
Cho et al (2009) [42]	Rat	Sprague-Dawley	M	IV	100	HPLC-UV	14.6	10.9	307
Lee et al (2010) [43]	Rat	Sprague-Dawley	M	IV	100	HPLC-UV	22.3	6.67	755
Choi et al (2010) [44]	Rat	Sprague-Dawley	M	IV	100	HPLC-UV	14.7	11.6	383
Choi and Lee (2012) [45]	Rat	Sprague-Dawley	M	IV	100	HPLC-UV	21.3	14.9	764
Lee et al (2013) [46]	Rat	Sprague-Dawley	M	IV	50	HPLC-UV	33.8	25.8	929
Kwon et al (2015) [47]	Rat	Sprague-Dawley	M	IV	2	LC-MS/MS	25.9	-	867
Ma et al (2016) [48]	Rat	Wistar	M	IV	25	HPLC-UV	8	28.6-74.2%	3250
			F	IV	25	HPLC-UV	6	26.7-57.6%	3530
Gabr et al (2017) [49]	Rat	Sprague-Dawley	M	IV	30	LC-MS	29.5	27.2	2320
Ma et al (2018) [50]	Rat	Wistar	M	IV	25	HPLC-UV	23.3	70.3%**	3050
Yang et al (2018) [51]	Rat	Wistar	M	IV	25	LC-MS/MS	9.07	7.05	1040

Nishizawa et al (2019) [52]	Rat	Wistar	M	IV	30	LC-MS/MS	41.2	23.0	1320
Han and Choi (2020) [53]	Rat	Sprague-Dawley	M	IV	30	HPLC-UV	24.2	16.1	349
Bouriche et al (2020) [54]*	Rabbit	New Zealand	F	IV	5	HPLC-UV	2.05	-	413
Michels et al (1999) [55]*	Cat	Domestic shorthair	-	IV	25	HPLC-UV	2.5	2.17	550
Shen et al (2016) [56]*	Monkey	Cynomolgus	M	IV	3.9	LC-MS/MS	11.2	10.7	980
Morse et al (2017) [57]	Monkey	Cynomolgus	M	IV	2.5	LC-MS/MS	8.06**	86%	353**
Patel et al (2017) [58]	Minipig	Yucatan	M	IV	0.5	LC-MS/MS	10.1	-	-
		Hanford*	M	IV	0.5	LC-MS/MS	9.7	-	2260
		Sinclair	M	IV	0.5	LC-MS/MS	8.7	-	991
		Gottingen	M	IV	0.5	LC-MS/MS	19.6	-	695
Johnston et al (2017) [59]*	Dog	Mixed	-	IV	24.8	FIA-MS/MS	24.1	-	10100
Sirtori et al (1978) [60]*	Man	Healthy	4 M 1 F	IV	926 mg	GC-MS	6.13	4.65	432**
Pentikäinen et al (1979) [61]*	Man	Healthy	1 M 2 F	IV	500 mg	LSC	7.61	7.52	856**
Tucker et al (1981) [62]*	Man	Healthy	M	IV	250 mg	GC-EC	10.1	7.83	511**
Hustace et al (2009) [63]*	Horse	-	-	IV	6 g	HPLC-UV	10.8	-	2250**

* Dataset used for minimal physiologically-based pharmacokinetic model fitting across 9 species
** Calculated from digitized data

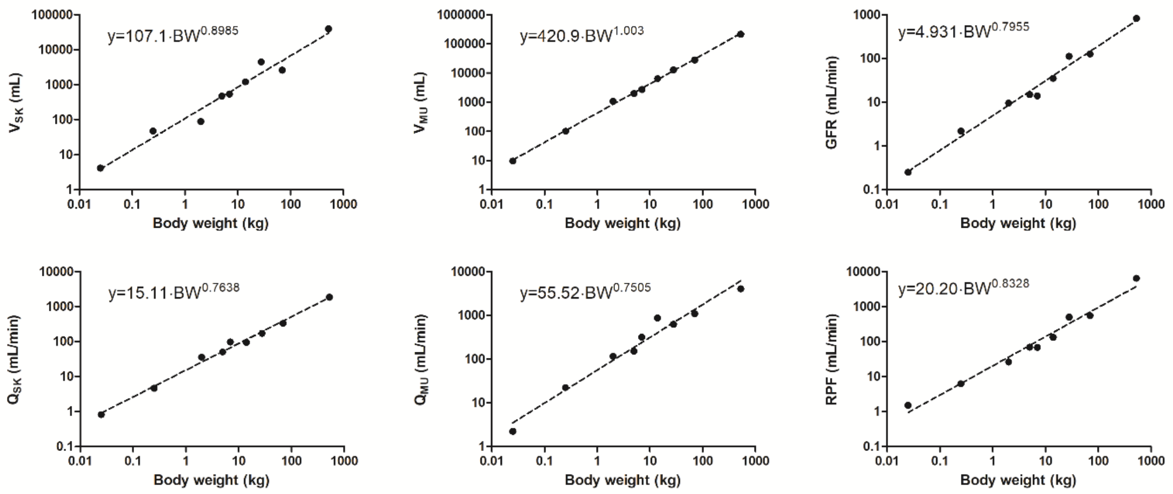

Supplementary Figure S1. Physiological and anatomical information (i.e., V_{SK}, V_{MU}, Q_{SK}, Q_{MU}, RPF, and GFR) collected from different sources (Supplementary Table S2) showed an allometric relationship among 9 species [i.e., mouse (0.025 kg), rat (0.25 kg), rabbit (2 kg), cat (5 kg), monkey (7 kg), minipig (14 kg), dog (28 kg), man (70 kg), and horse (530 kg)]

Supplementary Table S2. Summary of physiological input variables for meta-analysis of metformin pharmacokinetics in various species

	Mouse	Rat	Rabbit	Cat	Monkey	Minipig	Dog	Man	Horse
BW (kg)	0.025	0.25	2	5	7	14	28	70	530
V_B (mL)	1.64	15.3	120	300	420	875	2520	5200[a]	39800
V_{SK} (mL)	4.13	47.5	88[a]	469[b]	540[c]	1200[d]	4480	2600	39200
V_{MU} (mL)	9.6	101	1080[a]	1980[b]	2750[c]	6390	12800	28000	213000
Q_{CO} (mL/min)	14.0	80.0	395	786	1010	1700	2860	5690	26000
Q_{SK} (mL/min)	0.813	4.64	35.6[e]	50.4[b]	96.4[c]	94.8[d]	172	330	1840[f]
Q_{MU} (mL/min)	2.23	22.2	116[a]	151[b]	315[c]	863[d]	621	1090	4040[f]
GFR (mL/min)	0.25	2.18	9.6	15[g]	14	35[d]	112	126	820[h]
RPF (mL/min)	1.50[i]	6.20	26.1[e]	69.2[b]	67.3[c]	131[d]	500[j]	547	6400[k]
Gut radius (R) (cm)	0.135[l]	0.2	0.246[m]	0.35[n]	0.6	1[d]	1.25[d]	2.5	3[o]
V_{Lumen} (mL)	0.4	6.18	74.5	145[p]	894	754	1000	330	15500[p]
T_{SI} (min)	96.2[q]	103[r]	80[s]	144[t]	180	210	111	238	240[u]

V_{SK}, V_{MU}, Q_{SK}, Q_{MU}, RPF, and $Q_{CO}(= 0.235 \cdot BW^{0.75})$ from Brown et al (1997) [1], V_B from Wolfensohn & Lloyd (2003) [2], GFR from Lin (1995) [3], and gut radius from Kararli (1995) [4], unless otherwise noted

[a]Davies & Morris (1993) [5]; [b]Lindstedt & Schaeffer (2002) [6]; [c]Values adopted in Simcyp V19 (Simcyp Ltd. Sheffield, UK) [7];

[d]Suenderhauf & Parrott (2013) [8]; [e]Sweeny et al (2009) [9]; [f]Staddon et al (1984) [10]; [g]Von Hendy-Willson&Pressler (2011) [11]; [h]Walsh and Royal (1992) [12]; [i]Thuesen et al (2014) [13]; [j]Wesolowski et al (2019) [14]; [k]Holdstock et al (1998) [15]; l Ferraris et al (1989) [16]; [m]Merchant et al (2011) [17]; [n]Bettini et al (2003) [18]; [o]Clauss et al (2003) [19]

V_{Lumen} as the sum of fluid volume in stomach and small intestine, obtained from Hatton et al (2015) [20]; [p]cat and horse values were estimated by the interpolation and extrapolation from allometric relationship between V_{Lumen} and BW in the 7 species ($V_{Lumen} = 28.9 \cdot BW^{1.00}$, R^2=0.88)

T_{SI} is the small intestinal transit time obtained from Hatton et al (2015) [20]; [q]Myagmarjalbuu et al (2013) [21]; [r]Quini et al (2012) [22]; [s]Davies and Davies (2003) [23], considering jejunum and ileum; [t]Chandler et al (1997) [24]; [u]Steinmann et al (2020) [25]

Supplementary Table S3. Literature information collected for tissue distribution and blood partitioning of metformin

Species	Tissue	Value	Sex	Comments	Source
K_p					
Mouse	Liver	4.47	M	C_t/C_p (0.5 hr)	Wilcock and Bailey (1994) [64]
		4.95	M	C_t/C_p (1 hr)	Wilcock and Bailey (1994) [64]
		4.86	M	C_t/C_p (2 hr)	Wilcock and Bailey (1994) [64]
		7.10	M	C_t/C_p (4 hr)	Wilcock and Bailey (1994) [64]
		1.72	M	AUC_t/AUC_p (0-8 hr)	Lee et al (2014) [65]
		1.82	F	AUC_t/AUC_p (0-8 hr)	Lee et al (2014) [65]
		3.35	M	C_t/C_p (at day 7)	Chaudhari et al (2020) [66]
		3.69	F	C_t/C_p (at day 7)	Chaudhari et al (2020) [66]
		2.52	M	C_t/C_p (at day 30)	Chaudhari et al (2020) [66]
		3.90	F	C_t/C_p (at day 30)	Chaudhari et al (2020) [66]
		4.20	M	Median (10-300 mg/kg, 1.5-2.5 hr)	Higgins et al (2012) [27]*
		2.30	M	$C_{t,ss}/C_{p,ss}$	Ito et al (2012) [67]
		1.81	M	C_t/C_p (at day 98)	Chae et al (2019) [68]
		1.88	M	C_t/C_p (at 24 hr)	Toyama et al (2012) [69]*
		2.13	M	$C_{t,ss}/C_{p,ss}$	Nakamichi et al (2013) [28]*
		4.83	F	C_t/C_p (at 10 min)	Wang et al (2002) [70]*
	Brain	0.184	M	C_t/C_p (at day 7)	Chaudhari et al (2020) [66]
		0.257	F	C_t/C_p (at day 7)	Chaudhari et al (2020) [66]
		0.174	M	C_t/C_p (at day 30)	Chaudhari et al (2020) [66]
		0.237	F	C_t/C_p (at day 30)	Chaudhari et al (2020) [66]
		0.0354	M	$C_{t,ss}/C_{p,ss}$	Nakamichi et al (2013) [28]*
	Kidney	3.35	M	AUC_t/AUC_p (0-8 hr)	Lee et al (2014) [65]
		3.55	F	AUC_t/AUC_p (0-8 hr)	Lee et al (2014) [65]

	5.30	M	C_t/C_p (at day 7)	Chaudhari et al (2020) [66]
	6.64	F	C_t/C_p (at day 7)	Chaudhari et al (2020) [66]
	4.90	M	C_t/C_p (at day 30)	Chaudhari et al (2020) [66]
	6.44	F	C_t/C_p (at day 30)	Chaudhari et al (2020) [66]
	11.8	M	Median (10-300 mg/kg, 1.5-2.5 hr)	Higgins et al (2012) [27]*
	5.00	M	$C_{t,ss}/C_{p,ss}$	Ito et al (2012) [67]
	16.0	M	C_t/C_p (at day 98)	Chae et al (2019) [68]
	7.84	M	C_t/C_p (at 24 hr)	Toyama et al (2012) [69]*
	13.6	M	$C_{t,ss}/C_{p,ss}$	Nakamichi et al (2013) [28]*
	20.5	F	C_t/C_p (at 10 min)	Wang et al (2002) [70]*
Muscle	0.537	M	AUC_t/AUC_p (0-8 hr)	Lee et al (2014) [65]
	0.582	F	AUC_t/AUC_p (0-8 hr)	Lee et al (2014) [65]
	1.38	M	C_t/C_p (at day 7)	Chaudhari et al (2020) [66]
	2.06	F	C_t/C_p (at day 7)	Chaudhari et al (2020) [66]
	1.00	M	C_t/C_p (at day 30)	Chaudhari et al (2020) [66]
	1.59	F	C_t/C_p (at day 30)	Chaudhari et al (2020) [66]
	0.771	M	C_t/C_p (at 24 hr)	Toyama et al (2012) [69]*
	0.359	M	$C_{t,ss}/C_{p,ss}$	Nakamichi et al (2013) [28]*
Heart	0.599	M	AUC_t/AUC_p (0-8 hr)	Lee et al (2014) [65]
	0.712	F	AUC_t/AUC_p (0-8 hr)	Lee et al (2014) [65]
	0.519	M	$C_{t,ss}/C_{p,ss}$	Nakamichi et al (2013) [28]*
Adipose	0.471	M	$C_{t,ss}/C_{p,ss}$	Nakamichi et al (2013) [28]*
Stomach	5.25	M	C_t/C_p (at 0.5 hr)	Wilcock and Bailey (1994) [64]
	4.67	M	C_t/C_p (at 1 hr)	Wilcock and Bailey (1994) [64]
	6.57	M	C_t/C_p (at 2 hr)	Wilcock and Bailey (1994) [64]
	9.03	M	C_t/C_p (at 4 hr)	Wilcock and Bailey (1994) [64]

	Small intestine	13.7-15.3	M	C_t/C_p (at 0.5 hr)	Wilcock and Bailey (1994) [64]
		14.6-21.1	M	C_t/C_p (at 1 hr)	Wilcock and Bailey (1994) [64]
		9.57-21.3	M	C_t/C_p (at 2 hr)	Wilcock and Bailey (1994) [64]
		12.3-17.7	M	C_t/C_p (at 4 hr)	Wilcock and Bailey (1994) [64]
		4.42	M	$C_{t,ss}/C_{p,ss}$	Nakamichi et al (2013) [28]*
		0.837	F	C_t/C_p (at 10 min)	Wang et al (2002) [70]*
	Colon	4.52	M	C_t/C_p (at 0.5 hr)	Wilcock and Bailey (1994) [64]
		6.17	M	C_t/C_p (at 1 hr)	Wilcock and Bailey (1994) [64]
		6.29	M	C_t/C_p (at 2 hr)	Wilcock and Bailey (1994) [64]
		13.9	M	C_t/C_p (at 4 hr)	Wilcock and Bailey (1994) [64]
	Salivary gland	2.60	M	AUC_t/AUC_p (0-8 hr)	Lee et al (2014) [65]
		3.45	F	AUC_t/AUC_p (0-8 hr)	Lee et al (2014) [65]
Rat	Liver	2.91	M	C_t/C_p (at 2 hr)	Ma et al (2016) [71]*
		3.40	M	C_t/C_p (at 2 hr of day 7)	Ma et al (2016) [71]*
		3.04-3.47	M	C_t/C_p (at 1 hr)	You et al (2018) [72]
		3.09-3.53	M	C_t/C_p (at 3 hr)	You et al (2018) [72]
		0.368-0.502	M	C_t/C_p (at 12 hr)	You et al (2018) [72]
		6.83	M	C_t/C_p (at 2 hr)	Maeda et al (2007) [37]
		0.773	M	C_t/C_p (at 0.5 hr)	Han and Choi [53]
		2.43	M	C_t/C_p (at 1 hr)	Han and Choi [53]
		3.81	M	C_t/C_p (at 3 hr)	Han and Choi [53]
		3.50	M	C_t/C_p (at 6 hr)	Han and Choi [53]
		0.491	M	C_t/C_p (at 24 hr), diabetic rats	Wu et al (2019) [73]*
	Kidney	5.15	M	C_t/C_p (at 2 hr)	Ma et al (2016) [71]*
		5.84	M	C_t/C_p (at 2 hr of day 7)	Ma et al (2016) [71]*
		16.6	M	C_t/C_p (at 4 hr)	Nishizawa et al (2019) [52]

		4.16-5.92	M	C_t/C_p (at 1 hr)	You et al (2018) [72]
		4.86-5.64	M	C_t/C_p (at 3 hr)	You et al (2018) [72]
		0.604-0.861	M	C_t/C_p (at 12 hr)	You et al (2018) [72]
		24.9	M	C_t/C_p (at 2 hr)	Maeda et al (2007) [37]
		0.128	M	C_t/C_p (at 0.5 hr)	Han and Choi [53]
		3.39	M	C_t/C_p (at 1 hr)	Han and Choi [53]
		4.92	M	C_t/C_p (at 3 hr)	Han and Choi [53]
		5.92	M	C_t/C_p (at 6 hr)	Han and Choi [53]
		0.923	M	C_t/C_p (at 24 hr), diabetic rats	Wu et al (2019) [73]*
	Brain	0.2	M	C_t/C_p (at 1 hr)	Łabuzek et al (2010) [74]
		0.69	M	C_t/C_p (at 4 hr)	Łabuzek et al (2010) [74]
		0.99	M	C_t/C_p (at 6 hr)	Łabuzek et al (2010) [74]
		0.64	M	C_t/C_p (at 12 hr)	Łabuzek et al (2010) [74]
		1.48	M	C_t/C_p (at 24 hr)	Łabuzek et al (2010) [74]
	Heart	0.761	M	C_t/C_p (at 24 hr), diabetic rats	Wu et al (2019) [73]*
	Spleen	0.956	M	C_t/C_p (at 2 hr)	Maeda et al (2007) [37]
	Gut	4.63	M	C_t/C_p (at 2 hr)	Maeda et al (2007) [37]
		1.00	M	C_t/C_p (at 24 hr), diabetic rats	Wu et al (2019) [73]*
	Muscle	0.455	M	C_t/C_p (at 2 hr)	Ma et al (2016) [71]*
		0.738	M	C_t/C_p (at 2 hr of day 7)	Ma et al (2016) [71]*
		0.640	M	C_t/C_p (at 24 hr), diabetic rats	Wu et al (2019) [73]*
Monkey	Liver	15	M	Fitted (equivalent to AUC_t/AUC_p)	Morse et al (2017) [57]
$f_{d,tissue}$					
Mouse	Kidney	0.0953	M	Kidney slice	Ito et al (2012) [67]
Rat	Liver	0.231		In vitro hepatocytes	Umehara et al (2007) [75]
		0.345	M	In vivo	Kimura et al (2005) [76]*
		0.236	F	In vivo	Kimura et al (2005) [76]*
		0.261	M	In vivo	Jin et al (2009) [41]
		0.0172		In vitro hepatocytes	Liao et al (2019) [77]
	Kidney	0.443	M	In vivo	Kimura et al (2005) [76]*
		0.334	F	In vivo	Kimura et al (2005) [76]*
		0.00806	M	Kidney slice	Ma et al (2016) [71]*

		0.262	M	In vivo	Jin et al (2009) [41]
		0.996	M	Kidney slice	Umehara et al (2008) [78]
Monkey	Liver	0.0428		In vitro hepatocytes	Liao et al (2019) [77]
		0.0722	M	In vitro hepatocytes	Liao et al (2019) [77]
		0.0342	F	In vitro hepatocytes	Liao et al (2019) [77]
Dog	Liver	0.0250		In vitro hepatocytes	Liao et al (2019) [77]
Man	Liver	0.0269		In vitro hepatocytes	Umehara et al (2007) [75]
		0.0285		In vitro hepatocytes	Liao et al (2019) [77]
		0.0185	M	In vitro hepatocytes	Liao et al (2019) [77]
		0.0370	F	In vitro hepatocytes	Liao et al (2019) [77]
f_u					
Rat	Plasma	0.849		Equilibrium dialysis (10 µg/mL)	Choi et al (2006) [32]
		0.874		Equilibrium dialysis (5 µg/mL)	Choi and Lee (2012) [45]
		0.897		Equilibrium dialysis (0.1 – 200 µg/mL)	Choi et al (2010) [44]
Dog	Plasma	0.93		Centrifugal filtration (0.05 – 10 mM)	Garrett et al (1972) [79]
		0.83-0.951		Equilibrium dialysis (0.05 – 10 mM)	Garrett et al (1972) [79]
	Blood	0.92		Centrifugal filtration (0.05 – 10 mM)	Garrett et al (1972) [79]
Man	Plasma	1		Equilibrium dialysis (0.05 – 5 µg/mL)	Sirtori et al (1978) [60]
		1		Equilibrium dialysis (0.05 – 50 µg/mL)	Pentikäinen et al (1979) [61]
		1		Equilibrium dialysis (0.1 – 10 µg/mL)	Tucker et al (1981) [62]
		0.899		Centrifugal filtration (0.05 – 10 mM)	Garrett et al (1972) [79]
		0.75-0.98		Equilibrium dialysis (0.05 – 10 mM)	Garrett et al (1972) [79]
	Blood	0.932		Centrifugal filtration (0.05 – 10 mM)	Garrett et al (1972) [79]
R_b					
Rat		0.76-0.98		In vitro (0.1 – 10 µg/mL, 72 hr)	Xie et al (2015) [80]
		0.98-1.37		In vitro (0.1 – 10 µg/mL, 168 hr)	Xie et al (2015) [80]
Man		0.83-1.23		In vitro (0.1 – 10 µg/mL, 168 hr)	Xie et al (2015) [80]

*Calculated from digitized data

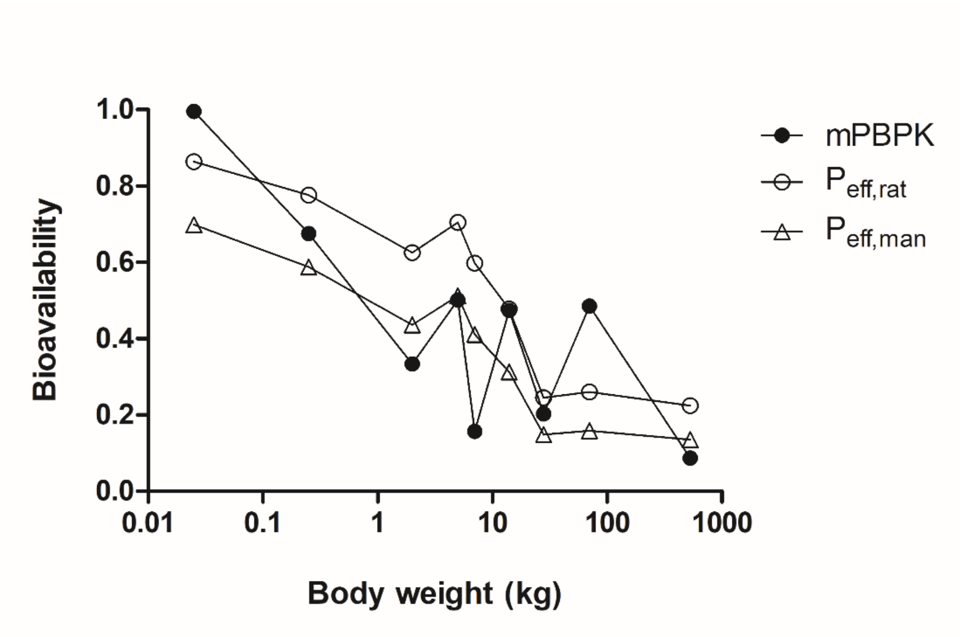

Supplementary Figure S2. ACAT model-based estimation of bioavailability using $F_a = 1 - \left(1 + 2P_{eff}T_{SI}/7R\right)^{-7}$ (7-enteric compartments), assuming that the effective intestinal permeability (P_{eff}) of the 9 species is the same with that of rat ($P_{eff,rat}$), and that of man ($P_{eff,man}$) predicted from Caco-2 cell permeability, which were compared with bioavailability determined from our mPBPK modeling (1-enteric compartment as a cylinder).

Supplementary Table S4. Calculation of the steady-state volume of distribution (V_{SS}) using *in vivo* tissue K_p values based on the equation $V_{SS} = V_B + \sum V_{T,i} K_{p,i}$ [81]. Tissue K_p values were assumed to be muscle K_p (1.03 for mouse and 0.597 for rat) if unavailable in Table 6.

**Tissue**	**Mouse (0.025 kg)**			**Tissue**	**Rat (0.25 kg)**		
	V_T **(mL)**	K_p	$V_T \cdot K_p$ **(mL)**		V_T **(mL)**	K_p	$V_T \cdot K_p$ **(mL)**
Adipose	1.88	0.471	0.885	Adipose	16.7	0.597	9.97
Bone	1.51	1.03	1.56	Bone	15.7	0.597	9.37
Brain	0.45	0.213	0.0959	Brain	1.24	0.8	0.992
Gut	2.56	11.3	28.9	Gut	6.19	4.63	28.7
Heart	0.12	0.610	0.0732	Heart	1.05	0.597	0.627
Kidney	0.34	8.74	2.97	Kidney	2.19	4.04	8.85
Liver	1.19	3.47	4.13	Liver	8.57	3.07	26.3
Lung	0.15	1.03	0.155	Lung	1.24	0.597	0.740
Muscle	9.5	1.03	9.79	Muscle	116	0.597	69.3
Skin	3.07	1.03	3.16	Skin	39.4	0.597	23.5
Spleen	0.11	1.03	0.113	Spleen	0.57	0.956	0.545
Blood (V_B)	1.64			Blood (V_B)	15.3		
		$V_{SS,mouse}$ **(mL/kg)**	**2140**			$V_{SS,rat}$ **(mL/kg)**	**777**

[a]V_T obtained from Simcyp V19

Supplementary Table S5. Model fitting results using a tri-exponential function [$C_p(t) = C_1e^{-\lambda_1 t} + C_2e^{-\lambda_2 t} + C_3e^{-\lambda_3 t}$] for metformin PK in various species (CV% obtained by fitting). It is noted that five parameters (C_1, λ_1, C_2, λ_2, and λ_3) were optimized while C_3 values were estimated as a secondary parameter ($C_3 = C_0 - C_1 - C_2$; $C_0 = Dose/V_B$)

**Species (body weight, kg)**	Source	C_0 (μg/mL)	C_1 (μg/mL)	λ_1 (min^{-1})	C_2 (μg/mL)	λ_2 (min^{-1})	C_3 (μg/mL)	λ_3 (min^{-1})	CL_D (mL/min)[a]	Q_{CO} (mL/min)[b]	CL (mL/min/kg)[c]	V_{SS} (mL/kg)[c]
Mouse (0.025)	Higgins et al (2012) [27]	76.2	69.1 (3.18)	2.49 (324)	6.52 (32.9)	0.197 (22.0)	0.601 (18.3)	0.0219 (12.0)	2.32 (455)	14.0	56.7 (97.4)	918 (189)
Rat (0.25)	Choi et al (2006) [32]	817	789 (0.404)	0.647 (5.56)	27.3 (11.5)	0.0414 (6.94)	1.01 (15.1)	0.00543 (8.21)	3.52 (10.7)	80.0	24.2 (3.25)	611 (6.79)
		1630	1550 (0.459)	0.572 (4.39)	80.6 (8.77)	0.0524 (4.54)	1.85 (8.78)	0.00547 (4.99)	2.90 (8.65)		21.8 (2.50)	456 (4.99)
		3270	2990 (1.49)	0.863 (19.7)	277 (16.0)	0.0494 (7.54)	3.85 (19.7)	0.00600 (8.49)	6.99 (29.7)		20.6 (6.90)	477 (12.2)
Rabbit (2)	Bouriche et al (2020) [54]	83.3	56.8 (6.48)	0.336 (17.5)	17.9 (18.7)	0.0389 (21.6)	8.63 (9.21)	0.00475 (5.63)	24.5 (15.4)	395	2.05 (1.87)	330 (3.72)
Cat (5)	Michels et al (1999) [55]	417	355 (1.31)	0.127 (7.88)	59.5 (7.58)	0.0114 (5.28)	2.17 (13.7)	0.00123 (11.3)	20.2 (11.0)	786	2.55 (2.43)	501 (8.43)
Monkey (7)	Shen et al (2016) [56]	65.0	62.6 (0.726)	0.210 (9.20)	2.35 (19.2)	0.0201 (11.5)	0.0422 (27.9)	0.00138 (9.61)	23.9 (19.9)	1010	8.75 (5.39)	576 (13.0)
Minipig (14)	Patel et al (2017) [58]	8.0	7.24 (2.15)	0.752 (21.2)	0.744 (20.8)	0.0250 (14.7)	0.0176 (21.8)	0.00122 (16.5)	468 (25.5)	1700	9.29 (7.69)	2240 (19.7)
Dog (28)	Johnston et al (2017) [59]	275	266 (0.175)	0.201 (4.66)	8.96 (5.15)	0.0123 (3.03)	0.217 (6.24)	0.00105 (4.47)	183 (8.33)	2860	10.9 (2.56)	1270 (5.65)
Man (70)	Tucker et al (1981) [62]	48.1	43.1 (1.47)	0.164 (6.91)	4.56 (13.0)	0.0175 (9.43)	0.404 (19.9)	0.00395 (8.68)	373 (11.1)	5690	5.71 (2.64)	387 (5.02)
	Pentikäinen et al (1979) [61]	96.1	79.7 (0.725)	0.930 (19.4)	13.5 (4.05)	0.0325 (4.21)	2.91 (4.10)	0.00658 (1.48)	3500 (21.5)		7.57 (1.62)	643 (3.00)
	Sirtori et al (1978) [60]	178	136 (7.01)	0.513 (36.4)	34.5 (25.8)	0.0391 (25.6)	7.40 (25.3)	0.00768 (9.25)	1640 (39.6)		6.27 (4.96)	441 (8.22)
Horse (530)	Hustace et al (2009) [63]	150	125 (4.32)	0.208 (28.0)	25.2 (21.5)	0.0297 (8.87)	0.119 (fixed)	0.000599 (fixed)	3460 (44.5)	26000	6.86 (8.54)	1510 (16.7)

[a] $CL_D = Dose\left(\frac{\sum C_i\lambda_i}{C_0^2} - \frac{1}{AUC}\right)$ estimated as a secondary parameter

[b] Q_{CO} obtained from Supplementary Table S1

[c] CL and V_{SS} estimated as a secondary parameter where $CL = \frac{Dose}{AUC}$ and $V_{SS} = \frac{Dose \cdot AUMC}{AUC^2}$

References

1. Brown, R.; Delp, M.; Lindstedt, S.; Rhomberg, L.; Beliles, R. Physiological parameter values for physiologically based pharmacokinetic models. *Toxicol. Ind. Health* **1997**, *13407*, 407-484.
2. Wolfensohn, S.; Lloyd, M. Handbook of laboratory animal management and welfare. **2003**.
3. Lin, J.H. Species similarities and differences in pharmacokinetics. *Drug Metab. Dispos.* **1995**, *23*, 1008-1021.
4. Kararli, T.T. Comparison of the gastrointestinal anatomy, physiology, and biochemistry of humans and commonly used laboratory animals. *Biopharm. Drug Dispos.* **1995**, *16*, 351-380, doi:10.1002/bdd.2510160502.
5. Davies, B.; Morris, T. Physiological parameters in laboratory animals and humans. *Pharm. Res.* **1993**, *10*, 1093-1095.
6. Lindstedt, S.L.; Schaeffer, P. Use of allometry in predicting anatomical and physiological parameters of mammals. *Lab. Anim.* **2002**, *36*, 1-19.
7. Jamei, M.; Marciniak, S.; Feng, K.; Barnett, A.; Tucker, G.; Rostami-Hodjegan, A. The Simcyp® population-based ADME simulator. *Expert Opin. Drug Metab. Toxicol.* **2009**, *5*, 211-223.
8. Suenderhauf, C.; Parrott, N. A physiologically based pharmacokinetic model of the minipig: data compilation and model implementation. *Pharm. Res.* **2013**, *30*, 1-15.
9. Sweeney, L.M.; Kirman, C.R.; Gannon, S.A.; Thrall, K.D.; Gargas, M.L.; Kinzell, J.H. Development of a physiologically based pharmacokinetic (PBPK) model for methyl iodide in rats, rabbits, and humans. *Inhalation Toxicol.* **2009**, *21*, 552-582, doi:10.1080/08958370802601569.
10. Staddon, G.; Weaver, B.; Lunn, C. A “standard horse” for use in physiologically based mathematical modelling. *Equine Vet. J.* **1984**, *16*, 189-191.
11. Von Hendy-Willson, V.E.; Pressler, B.M. An overview of glomerular filtration rate testing in dogs and cats. *Vet. J.* **2011**, *188*, 156-165.
12. Walsh, D.; Royal, H. Evaluation of a single injection of 99mTc-labeled diethylenetriaminepentaacetic acid for measuring glomerular filtration rate in horses. *Am. J. Vet. Res.* **1992**, *53*, 776-780.
13. Thuesen, A.D.; Andersen, H.; Cardel, M.; Toft, A.; Walter, S.; Marcussen, N.; Jensen, B.L.; Bie, P.; Hansen, P.B. Differential effect of T-type voltage-gated Ca2+ channel disruption on renal plasma flow and glomerular filtration rate in vivo. *Am. J. Physiol.: Renal, Fluid Electrolyte Physiol.* **2014**, *307*, F445-F452.
14. Wesolowski, C.A.; Wanasundara, S.N.; Babyn, P.S.; Alcorn, J. Comparison of the gamma-Pareto convolution with conventional methods of characterising metformin pharmacokinetics in dogs. *J. Pharmacokinet. Pharmacodyn.* **2020**, *47*, 19-45.
15. Holdstock, N.B.; Ousey, J.C.; Rossdale, P. Glomerular filtration rate, effective renal plasma flow, blood pressure and pulse rate in the equine neonate during the first 10 days post partum. *Equine Vet. J.* **1998**, *30*, 335-343.
16. Ferraris, R.P.; Lee, P.P.; Diamond, J.M. Origin of regional and species differences in intestinal glucose uptake. *Am. J. Physiol.: Gastrointest. Liver Physiol.* **1989**, *257*, G689-G697.
17. Merchant, H.A.; McConnell, E.L.; Liu, F.; Ramaswamy, C.; Kulkarni, R.P.; Basit, A.W.; Murdan, S. Assessment of gastrointestinal pH, fluid and lymphoid tissue in the guinea pig, rabbit and pig, and implications for their use in drug development. *Eur. J. Pharm. Sci.* **2011**, *42*, 3-10.
18. Bettini, G.; Muracchini, M.; Della Salda, L.; Preziosi, R.; Morini, M.; Guglielmini, C.; Sanguinetti, V.; Marcato, P. Hypertrophy of intestinal smooth muscle in cats. *Res. Vet. Sci.* **2003**, *75*, 43-53.
19. Clauss, M.; Frey, R.; Kiefer, B.; Lechner-Doll, M.; Loehlein, W.; Polster, C.; Rössner, G.; Streich, W.J. The maximum attainable body size of herbivorous mammals: morphophysiological constraints on foregut, and adaptations of hindgut fermenters. *Oecologia* **2003**, *136*, 14-27.
20. Hatton, G.B.; Yadav, V.; Basit, A.W.; Merchant, H.A. Animal farm: considerations in animal gastrointestinal physiology and relevance to drug delivery in humans. *J. Pharm. Sci.* **2015**, *104*, 2747-2776.
21. Myagmarjalbuu, B.; Moon, M.J.; Heo, S.H.; Jeong, S.I.; Park, J.-S.; Jun, J.Y.; Jeong, Y.Y.; Kang, H.K. Establishment of a protocol for determining gastrointestinal transit time in mice using barium and radiopaque markers. *Korean J. Radiol.* **2013**, *14*, 45.
22. Quini, C.C.; Américo, M.F.; Corá, L.A.; Calabresi, M.F.; Alvarez, M.; Oliveira, R.B.; Miranda, J.A. Employment of a noninvasive magnetic method for evaluation of gastrointestinal transit in rats. *J. Biol. Eng.* **2012**, *6*, 1-6.
23. Davies, R.R.; Davies, J.A.R. Rabbit gastrointestinal physiology. *Vet. Clin. North Am. Exot. Anim. Pract.* **2003**, *6*, 139-153.

24. Chandler, M.L.; Guilford, G.; Lawoko, C.R. Radiopaque markers to evaluate gastric emptying and small intestinal transit time in healthy cats. *J. Vet. Intern. Med.* **1997**, *11*, 361-364.
25. Steinmann, M.; Bezugley, R.J.; Bond, S.L.; Pomrantz, J.S.; Léguillette, R. A wireless endoscopy capsule suitable for imaging of the equine stomach and small intestine. *J. Vet. Intern. Med.* **2020**, *34*, 1622-1630.
26. Tsuda, M.; Terada, T.; Mizuno, T.; Katsura, T.; Shimakura, J.; Inui, K.-i. Targeted disruption of the multidrug and toxin extrusion 1 (mate1) gene in mice reduces renal secretion of metformin. *Mol. Pharmacol.* **2009**, *75*, 1280-1286.
27. Higgins, J.W.; Bedwell, D.W.; Zamek-Gliszczynski, M.J. Ablation of both organic cation transporter (OCT) 1 and OCT2 alters metformin pharmacokinetics but has no effect on tissue drug exposure and pharmacodynamics. *Drug Metab. Dispos.* **2012**, *40*, 1170-1177.
28. Nakamichi, N.; Shima, H.; Asano, S.; Ishimoto, T.; Sugiura, T.; Matsubara, K.; Kusuhara, H.; Sugiyama, Y.; Sai, Y.; Miyamoto, K.-i. Involvement of carnitine/organic cation transporter OCTN1/SLC22A4 in gastrointestinal absorption of metformin. *J. Pharm. Sci.* **2013**, *102*, 3407-3417.
29. Chen, E.C.; Liang, X.; Yee, S.W.; Geier, E.G.; Stocker, S.L.; Chen, L.; Giacomini, K.M. Targeted disruption of organic cation transporter 3 attenuates the pharmacologic response to metformin. *Mol. Pharmacol.* **2015**, *88*, 75-83.
30. Shirasaka, Y.; Lee, N.; Zha, W.; Wagner, D.; Wang, J. Involvement of organic cation transporter 3 (Oct3/Slc22a3) in the bioavailability and pharmacokinetics of antidiabetic metformin in mice. *Drug Metab. Pharmacokinet.* **2016**, *31*, 385-388.
31. KAKEMI, M.; SASAKI, H.; SAEKI, K.; ENDOH, M.; KATAYAMA, K.; KOIZUMI, T. Pharmacologic effects of metformin in relation to its disposition in alloxan diabetic rats. *J. Pharmacobio-Dyn.* **1983**, *6*, 71-87.
32. Choi, Y.H.; Kim, S.G.; Lee, M.G. Dose-independent pharmacokinetics of metformin in rats: Hepatic and gastrointestinal first-pass effects. *J. Pharm. Sci.* **2006**, *95*, 2543-2552, doi:10.1002/jps.20744.
33. Choi, Y.; Lee, M. Effects of enzyme inducers and inhibitors on the pharmacokinetics of metformin in rats: involvement of CYP2C11, 2D1 and 3A1/2 for the metabolism of metformin. *Br. J. Pharmacol.* **2006**, *149*, 424-430.
34. Choi, Y.H.; Lee, I.; Lee, M.G. Effects of bacterial lipopolysaccharide on the pharmacokinetics of metformin in rats. *Int. J. Pharm.* **2007**, *337*, 194-201.
35. Choi, Y.H.; Lee, I.; Lee, M.G. Effects of water deprivation on the pharmacokinetics of metformin in rats. *Biopharm. Drug Dispos.* **2007**, *28*, 373-383.
36. Choi, M.K.; Jin, Q.R.; Jin, H.E.; Shim, C.K.; Cho, D.Y.; Shin, J.G.; Song, I.S. Effects of tetraalkylammonium compounds with different affinities for organic cation transporters on the pharmacokinetics of metformin. *Biopharm. Drug Dispos.* **2007**, *28*, 501-510.
37. Maeda, T.; Oyabu, M.; Yotsumoto, T.; Higashi, R.; Nagata, K.; Yamazoe, Y.; Tamai, I. Effect of pregnane X receptor ligand on pharmacokinetics of substrates of organic cation transporter Oct1 in rats. *Drug Metab. Dispos.* **2007**, *35*, 1580-1586.
38. Lee, M.G.; Choi, Y.H.; Lee, I. Effects of diabetes mellitus induced by alloxan on the pharmacokinetics of metformin in rats: restoration of pharmacokinetic parameters to the control state by insulin treatment. *J. Pharm. Pharm. Sci.* **2008**, *11*, 88-103.
39. Choi, Y.H.; Lee, D.C.; Lee, I.; Lee, M.G. Changes in metformin pharmacokinetics after intravenous and oral administration to rats with short-term and long-term diabetes induced by streptozotocin. *J. Pharm. Sci.* **2008**, *97*, 5363-5375.
40. Choi, Y.; Chung, S.; Lee, M. Pharmacokinetic interaction between DA-8159, a new erectogenic, and metformin in rats: competitive inhibition of metabolism via hepatic CYP3A1/2. *Br. J. Pharmacol.* **2008**, *153*, 1568-1578.
41. Jin, H.-E.; Hong, S.-S.; Choi, M.-K.; Maeng, H.-J.; Kim, D.-D.; Chung, S.-J.; Shim, C.-K. Reduced antidiabetic effect of metformin and down-regulation of hepatic Oct1 in rats with ethynylestradiol-induced cholestasis. *Pharm. Res.* **2009**, *26*, 549-559.
42. Cho, Y.; Choi, Y.; Kim, S.; Lee, M. Effects of Escherichia coli lipopolysaccharide on the metformin pharmacokinetics in rats. *Xenobiotica; the fate of foreign compounds in biological systems* **2009**, *39*, 946-954.
43. Lee, J.; Kang, H.; Lee, M. Pharmacokinetic interaction between telithromycin and metformin in diabetes mellitus rats. *Xenobiotica; the fate of foreign compounds in biological systems* **2010**, *40*, 217-224.
44. Choi, Y.; Lee, U.; Lee, B.; Lee, M. Pharmacokinetic interaction between itraconazole and metformin in rats: competitive inhibition of metabolism of each drug by each other via hepatic and intestinal CYP3A1/2. *Br. J. Pharmacol.* **2010**, *161*, 815-829.

45. Choi, Y.H.; Lee, M.G. Pharmacokinetic and pharmacodynamic interaction between nifedipine and metformin in rats: competitive inhibition for metabolism of nifedipine and metformin by each other via CYP isozymes. *Xenobiotica; the fate of foreign compounds in biological systems* **2012**, *42*, 483-495.
46. Lee, Y.K.; Chin, Y.-W.; Choi, Y.H. Effects of Korean red ginseng extract on acute renal failure induced by gentamicin and pharmacokinetic changes by metformin in rats. *Food Chem. Toxicol.* **2013**, *59*, 153-159.
47. Kwon, M.; Choi, Y.A.; Choi, M.-K.; Song, I.-S. Organic cation transporter-mediated drug–drug interaction potential between berberine and metformin. *Arch. Pharmacal Res.* **2015**, *38*, 849-856.
48. Ma, Y.-r.; Qin, H.-y.; Jin, Y.-w.; Huang, J.; Han, M.; Wang, X.-d.; Zhang, G.-q.; Zhou, Y.; Rao, Z.; Wu, X.-a. Gender-related differences in the expression of organic cation transporter 2 and its role in urinary excretion of metformin in rats. *Eur. J. Drug Metab. Pharmacokinet.* **2016**, *41*, 559-565.
49. Gabr, R.Q.; El-Sherbeni, A.A.; Ben-Eltriki, M.; El-Kadi, A.O.; Brocks, D.R. Pharmacokinetics of metformin in the rat: assessment of the effect of hyperlipidemia and evidence for its metabolism to guanylurea. *Can. J. Physiol. Pharmacol.* **2017**, *95*, 530-538.
50. Ma, Y.-r.; Zhou, Y.; Huang, J.; Qin, H.-y.; Wang, P.; Wu, X.-a. The urinary excretion of metformin, ceftizoxime and ofloxacin in high serum creatinine rats: Can creatinine predict renal tubular elimination? *Life Sci.* **2018**, *196*, 110-117.
51. Yang, S.; Dai, Y.; Liu, Z.; Wang, C.; Meng, Q.; Huo, X.; Sun, H.; Ma, X.; Peng, J.; Liu, K. Involvement of organic cation transporter 2 in the metformin-associated increased lactate levels caused by contrast-induced nephropathy. *Biomed. Pharmacother.* **2018**, *106*, 1760-1766.
52. Nishizawa, K.; Yoda, N.; Morokado, F.; Komori, H.; Nakanishi, T.; Tamai, I. Changes of drug pharmacokinetics mediated by downregulation of kidney organic cation transporters Mate1 and Oct2 in a rat model of hyperuricemia. *PLoS One* **2019**, *14*, e0214862.
53. Han, S.Y.; Choi, Y.H. Pharmacokinetic interaction between metformin and verapamil in rats: Inhibition of the OCT2-mediated renal excretion of metformin by verapamil. *Pharmaceutics* **2020**, *12*, 468.
54. Bouriche, S.; Alonso-García, A.; Cárceles-Rodríguez, C.M.; Rezgui, F.; Fernández-Varón, E. Potential of sustained release microparticles of metformin in veterinary medicine: An in vivo pharmacokinetic study of metformin microparticles as oral sustained release formulation in rabbits. *BMC Vet. Res.* **2020**.
55. Michels, G.M.; Boudinot, F.; Ferguson, D.C.; Hoenig, M. Pharmacokinetics of the antihyperglycemic agent metformin in cats. *Am. J. Vet. Res.* **1999**, *60*, 738-742.
56. Shen, H.; Liu, T.; Jiang, H.; Titsch, C.; Taylor, K.; Kandoussi, H.; Qiu, X.; Chen, C.; Sukrutharaj, S.; Kuit, K. Cynomolgus monkey as a clinically relevant model to study transport involving renal organic cation transporters: in vitro and in vivo evaluation. *Drug Metab. Dispos.* **2016**, *44*, 238-249.
57. Morse, B.L.; MacGuire, J.G.; Marino, A.M.; Zhao, Y.; Fox, M.; Zhang, Y.; Shen, H.; Humphreys, W.G.; Marathe, P.; Lai, Y. Physiologically based pharmacokinetic modeling of transporter-mediated hepatic clearance and liver partitioning of OATP and OCT substrates in cynomolgus monkeys. *AAPS J.* **2017**, *19*, 1878-1889.
58. Patel, N.J.; Yumibe, N.; Ruterbories, K.; Huang, N.; Burns, L.; Tan, J.; White, D.; Liu, J.; Brocksmith, D.; Bouchard, G. Pharmacokinetics of intravenous and oral metformin and r, s-verapamil in Sinclair, Hanford, Yucatan and Göttingen minipigs. *Int. J. Pharmacokinet.* **2017**, *2*, 81-91.
59. Johnston, C.A.; Dickinson, V.S.M.; Alcorn, J.; Gaunt, M.C. Pharmacokinetics and oral bioavailability of metformin hydrochloride in healthy mixed-breed dogs. *Am. J. Vet. Res.* **2017**, *78*, 1193-1199.
60. Sirtori, C.R.; Franceschini, G.; Galli-Kienle, M.; Cighetti, G.; Galli, G.; Bondioli, A.; Conti, F. Disposition of metformin (N, N-dimethylbiguanide) in man. *Clin. Pharmacol. Ther.* **1978**, *24*, 683-693.
61. Pentikäinen, P.; Neuvonen, P.; Penttilä, A. Pharmacokinetics of metformin after intravenous and oral administration to man. *Eur. J. Clin. Pharmacol.* **1979**, *16*, 195-202.
62. Tucker, G.; Casey, C.; Phillips, P.; Connor, H.; Ward, J.; Woods, H. Metformin kinetics in healthy subjects and in patients with diabetes mellitus. *Br. J. Clin. Pharmacol.* **1981**, *12*, 235-246.
63. Hustace, J.L.; Firshman, A.M.; Mata, J.E. Pharmacokinetics and bioavailability of metformin in horses. *Am. J. Vet. Res.* **2009**, *70*, 665-668.
64. Wilcock, C.; Bailey, C. Accumulation of metformin by tissues of the normal and diabetic mouse. *Xenobiotica; the fate of foreign compounds in biological systems* **1994**, *24*, 49-57.
65. Lee, N.; Duan, H.; Hebert, M.F.; Liang, C.J.; Rice, K.M.; Wang, J. Taste of a pill. *J. Biol. Chem.* **2014**, *289*, 27055-27064.

66. Chaudhari, K.; Wang, J.; Xu, Y.; Winters, A.; Wang, L.; Dong, X.; Cheng, E.Y.; Liu, R.; Yang, S.-H. Determination of metformin bio-distribution by LC-MS/MS in mice treated with a clinically relevant paradigm. *PLoS One* **2020**, *15*, e0234571.
67. Ito, S.; Kusuhara, H.; Yokochi, M.; Toyoshima, J.; Inoue, K.; Yuasa, H.; Sugiyama, Y. Competitive inhibition of the luminal efflux by multidrug and toxin extrusions, but not basolateral uptake by organic cation transporter 2, is the likely mechanism underlying the pharmacokinetic drug-drug interactions caused by cimetidine in the kidney. *J. Pharmacol. Exp. Ther.* **2012**, *340*, 393-403.
68. Chae, H.-S.; You, B.H.; Choi, J.; Chin, Y.-W.; Kim, H.; Choi, H.S.; Choi, Y.H. Ginseng berry extract enhances metformin efficacy against obesity and hepatic steatosis in mice fed high-fat diet through increase of metformin uptake in liver. *J. Funct. Foods* **2019**, *62*, 103551.
69. Toyama, K.; Yonezawa, A.; Masuda, S.; Osawa, R.; Hosokawa, M.; Fujimoto, S.; Inagaki, N.; Inui, K.; Katsura, T. Loss of multidrug and toxin extrusion 1 (MATE1) is associated with metformin-induced lactic acidosis. *Br. J. Pharmacol.* **2012**, *166*, 1183-1191.
70. Wang, D.-S.; Jonker, J.W.; Kato, Y.; Kusuhara, H.; Schinkel, A.H.; Sugiyama, Y. Involvement of organic cation transporter 1 in hepatic and intestinal distribution of metformin. *J. Pharmacol. Exp. Ther.* **2002**, *302*, 510-515.
71. Ma, Y.r.; Shi, A.x.; Qin, H.y.; Zhang, T.; Wu, Y.f.; Zhang, G.q.; Wu, X.a. Metoprolol decreases the plasma exposure of metformin via the induction of liver, kidney and muscle uptake in rats. *Biopharm. Drug Dispos.* **2016**, *37*, 511-521.
72. You, B.H.; Chin, Y.W.; Kim, H.; Choi, H.S.; Choi, Y.H. Houttuynia cordata extract increased systemic exposure and liver concentrations of metformin through OCTs and MATEs in rats. *Phytother. Res.* **2018**, *32*, 1004-1013.
73. Wu, B.; Chen, M.; Gao, Y.; Hu, J.; Liu, M.; Zhang, W.; Huang, W. In vivo pharmacodynamic and pharmacokinetic effects of metformin mediated by the gut microbiota in rats. *Life Sci.* **2019**, *226*, 185-192.
74. Łabuzek, K.; Suchy, D.; Gabryel, B.; Bielecka, A.; Liber, S.; Okopień, B. Quantification of metformin by the HPLC method in brain regions, cerebrospinal fluid and plasma of rats treated with lipopolysaccharide. *Pharmacol. Rep.* **2010**, *62*, 956-965.
75. Umehara, K.-I.; Iwatsubo, T.; Noguchi, K.; Kamimura, H. Functional involvement of organic cation transporter1 (OCT1/Oct1) in the hepatic uptake of organic cations in humans and rats. *Xenobiotica; the fate of foreign compounds in biological systems* **2007**, *37*, 818-831.
76. Kimura, N.; Masuda, S.; Tanihara, Y.; Ueo, H.; Okuda, M.; Katsura, T.; Inui, K.-i. Metformin is a superior substrate for renal organic cation transporter OCT2 rather than hepatic OCT1. *Drug Metab. Pharmacokinet.* **2005**, *20*, 379-386.
77. Liao, M.; Zhu, Q.; Zhu, A.; Gemski, C.; Ma, B.; Guan, E.; Li, A.P.; Xiao, G.; Xia, C.Q. Comparison of uptake transporter functions in hepatocytes in different species to determine the optimal model for evaluating drug transporter activities in humans. *Xenobiotica; the fate of foreign compounds in biological systems* **2019**, *49*, 852-862.
78. Umehara, K.; Iwatsubo, T.; Noguchi, K.; Kamimura, H. Functional involvement of the organic cation transporter 2 (rOct2) in the renal uptake of organic cations in rats. *J. Int. Med. Res.* **2008**, *36*, 123-136.
79. Garrett, E.R.; Tsau, J.; Hinderling, P.H. Application of ion-pair methods to drug extraction from biological fluids II: Quantitative determination of biguanides in biological fluids and comparison of protein binding estimates. *J. Pharm. Sci.* **1972**, *61*, 1411-1418.
80. Xie, F.; Ke, A.B.; Bowers, G.D.; Zamek-Gliszczynski, M.J. Metformin's intrinsic blood-to-plasma partition ratio (B/P): reconciling the perceived high in vivo B/P> 10 with the in vitro equilibrium value of unity. *J. Pharmacol. Exp. Ther.* **2015**, *354*, 225-229.
81. Øie, S.; Tozer, T.N. Effect of altered plasma protein binding on apparent volume of distribution. *J. Pharm. Sci.* **1979**, *68*, 1203-1205.
